# P3MaZe: a Mass-Zero Constrained-Dynamics Formulation of Particle–Mesh Electrostatics

**DOI:** 10.1021/acs.jctc.6c01315

**Published:** 2026-10-26

**Authors:** Federica Troni, Violette Gontran, Davide Grassano, Sara Bonella

**Affiliations:** † Centre Européen de Calcul Atomique et Moléculaire (CECAM), Ecole Polytechnique Fédérale de Lausanne, 1015 Lausanne, Switzerland; ‡ National Centre for Computational Design and Discovery of Novel Materials (MARVEL), Ecole Polytechnique Fédérale de Lausanne, CH-1015 Lausanne, Switzerland

## Abstract

We introduce P3MaZe, a real-space particle–mesh electrostatic method that combines the standard short-range/long-range decomposition of Particle–Particle Particle–Mesh (P3M) electrostatics with the Mass-Zero constrained dynamics (MaZe) framework. In this formulation, the smooth long-range electrostatic potential is represented on a mesh as a zero-inertia auxiliary field, while the discretized Poisson equation is enforced as a holonomic constraint during molecular dynamics. By retaining the standard P3M decomposition, P3MaZe preserves the systematic accuracy controls associated with the real-space cutoff, the Ewald splitting, the mesh spacing, and the charge assignment procedure, while replacing the conventional multigrid Poisson solver by a constrained correction problem. The method is validated for molten NaCl and simple point-charge flexible water (SPC/Fw). Values of the potential, structural, translational, collective, and rotational dynamical observables are in quantitative agreement with those obtained with established electrostatic methods, including real-space P3M, and Ewald summation. We further show that, for the linear, time-independent Poisson operator considered here, the constrained correction problem is algebraically equivalent to a direct Poisson solve initialized by a chronological extrapolation of the mesh potential, a connection not previously established for MaZe-based approaches. This result identifies the precise mechanism through which acceleration of the iterative long-range solve is achieved, and we exploit it systematically through higher-order predictors to further improve convergence, while retaining the expected linear scaling with system size. Because the underlying extrapolation is independent of the constrained-dynamics formalism, it can, of course, also be used directly to accelerate conventional iterative real-space Poisson solvers.

## Graphical abstract

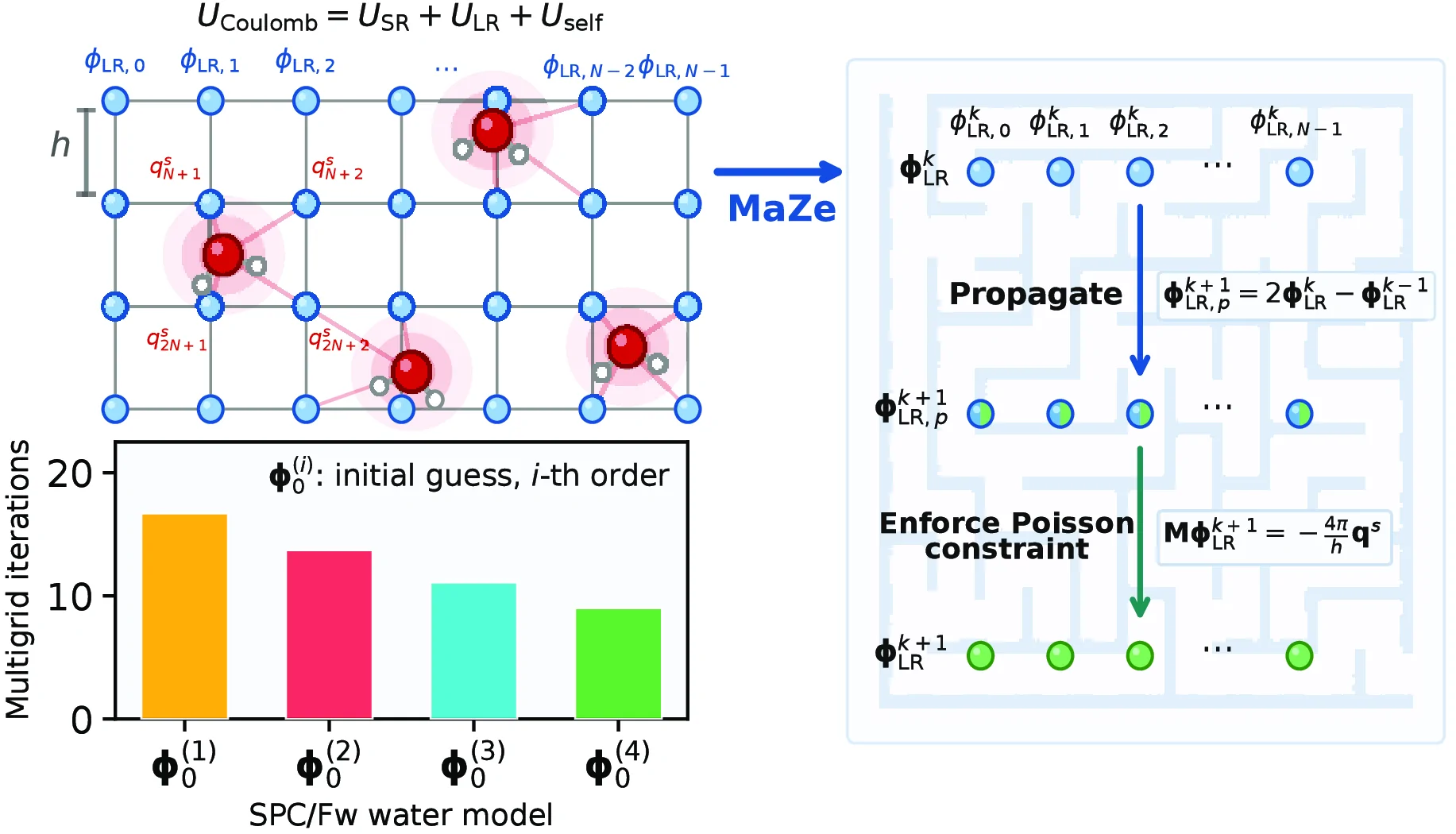

## Introduction

The evaluation of long-range electrostatic interactions remains one of the main challenges in large-scale classical molecular dynamics (MD) simulations. Unlike short-ranged van der Waals interactions, these interactions cannot be treated by a simple local truncation in a controlled manner.^1^ Under periodic boundary conditions (PBC), the lattice sum is conditionally convergent, and the electrostatic response contains both a singular short-range component, dominated by local pairwise interactions, and a long-range component that remains collective over the periodic system.^2^ Highly efficient methods have been developed to address this problem and are now routinely employed in molecular simulations.^3^ Nevertheless, when simulations scale to millions of degrees of freedom, as required for realistic modeling of complex biophysical and materials systems, long-range electrostatics continues to be one of the dominant computational bottlenecks.^4,5^ On modern heterogeneous many-core and GPU architectures, this cost is increasingly dominated by communication rather than arithmetic operations, motivating the development of algorithms with improved communication locality.^6–8^

The separation of length scales in the electrostatic response is at the heart of state-of-the-art algorithms that treat the short- and long-range components with different numerical approaches. This strategy forms the basis of Ewald-type methods and their particle-mesh variants.^9^ In Ewald summation, the Coulomb interaction is decomposed into a short-range contribution, evaluated by direct pair summation in real space, and a smooth long-range contribution, evaluated analytically in reciprocal space.^10^ Particle–mesh methods, including Particle Mesh Ewald^11,12^ and P3M,^13^ retain the Ewald short/long decomposition, but accelerate the smooth long-range calculation by solving it numerically. In reciprocal space, the discretized Poisson equation becomes diagonal and can therefore be solved efficiently using three-dimensional Fast Fourier transform (FFT). The accuracy of these methods can be systematically controlled through the real-space cutoff, the Ewald splitting parameter, the mesh spacing, and the charge-assignment order, which together determine the balance between real-space truncation error and mesh/discretization error. Particle–mesh methods combine systematic control of electrostatic accuracy with excellent computational efficiency and have consequently become the standard approach for large-scale molecular simulations. Although substantial effort has been devoted to improving the scalability of distributed FFT implementations in modern molecular simulation packages,^14–16^ the associated three-dimensional FFTs require global communication patterns that still limit scalability.^5–7^ Attempts to improve long-range solvers can be broadly divided into two classes: methods that avoid FFTs by adopting a different electrostatic formulation, and methods that retain the particle–mesh decomposition but replace the FFT-based mesh solver by a real-space one. The first class includes, for example, fast multipole and tree-based methods that replace the particle-mesh solve by hierarchical expansions of distant charge distributions.^17–19^ This can provide favorable scaling, but the accuracy is controlled by expansion order, clustering criteria, and the treatment of periodic images, rather than by the standard P3M parameters of splitting, mesh spacing, and charge assignment. A different approach is provided by multilevel summation methods. These retain a separation between local interactions and smooth corrections, but the long-range contribution is distributed over a hierarchy of grids, leading to a different interpolation and error structure from a single P3M mesh solve.^20,21^ An alternative strategy is adopted by local-field methods, such as those introduced by Maggs and Rossetto, which reformulate electrostatics in terms of locally evolving electric fields satisfying Gauss’s law.^22,23^ These methods eliminate global FFTs but depart from the standard particle–mesh decomposition and therefore adopt a fundamentally different numerical framework. Finally, more local alternatives (e.g., damped or shifted pairwise approximations) modify the Coulomb potential itself, thus avoiding a global solve. The quality of these approximations depends, however, on the system and on the observables considered.^24^

For the present work, the relevant route is the second one: retaining the P3M short/long decomposition and its associated error controls, while replacing the FFT-based mesh solve by a real-space solver. Within the class of P3M-based methods, Beckers et al. employed successive over-relaxation to solve the discretized Poisson equation on a real-space grid,^25^ while Sagui and Darden adopted a multigrid solver,^26^ achieving linear scaling with the number of particles and grid points. Beckers et al. noted that the converged solution from a previous configuration can serve as an effective initial guess for the subsequent iterative solve, but this observation was not developed further, and the multigrid approach of Sagui and Darden does not exploit an analogous initialization strategy. Related ideas have also been developed for nonperiodic systems. In particular, Sutmann and Steffen proposed a particle–particle particle–multigrid method in which the near-field contribution is evaluated explicitly and the far-field contribution is obtained from a multigrid Poisson solver with boundary conditions determined by a multipole expansion.^27^ These approaches demonstrate that replacing the FFT-based mesh solve is compatible with retaining the particle–mesh decomposition and its systematic accuracy control, motivating the search for more efficient real-space formulations.

In this paper, we introduce P3MaZe, a MaZe formulation of particle–mesh electrostatics. The MaZe formalism promotes appropriate auxiliary quantities, typically featuring as parameters subjected to conditions in the evolution of physical degrees of freedom, to zero-inertia dynamical variables whose values are determined by enforcing appropriate holonomic constraints during the dynamics. In this way, repeated solution of auxiliary optimization or field equations is replaced by constrained dynamical evolution while preserving the desired equilibrium distribution.

This approach was first proposed by Ciccotti and Ryckaert to describe rotation–translation coupling in diatomic molecules.^28^ Over the past few years, it has been revived and generalized to a broad range of problems, including classical polarizable models,^29,30^ constant-potential electrochemical simulations,^31^ shell models in magnetic fields,^32^ orbital-free first-principles MD,^33^ and advanced classical simulations of supercapacitors.^34^

In very recent work,^35^ the MaZe formalism was applied to the solution of electrostatic interactions under the name Poisson MaZe. In Poisson MaZe, the electrostatic potential on a real-space grid is represented by zero-inertia auxiliary variables, while the discretized Poisson equation is enforced as a holonomic constraint. An efficient multigrid constraint solver yields linear scaling with the number of degrees of freedom and improves the efficiency of the iterative electrostatic solver. The formulation also preserves key physical properties, including energy and momentum conservation, time reversibility, and the stationarity of ensemble averages. However, Poisson MaZe treats the full Coulomb problem on a grid. Consequently, the fine spatial resolution required to represent the short-range electrostatic potential must be maintained throughout the simulation domain, increasing both the number of auxiliary variables and the size of the constrained problem.

The present work overcomes these limitations by combining the systematic accuracy framework of particle–mesh electrostatics with the MaZe constrained-dynamics formalism. In P3MaZe, the short-range contribution is evaluated by conventional particle–particle summation, while only the smooth long-range mesh potential is represented by zero-inertia auxiliary variables. The corresponding mesh equation is enforced as a holonomic constraint at every time step, replacing the conventional FFT-based reciprocal-space solve by a local constrained solver. The method preserves systematic control of accuracy. Beyond the formulation itself, we present a previously unrecognized property of the MaZe dynamics: for the linear, time-independent Poisson operator considered here, the constrained correction problem is exactly equivalent to a direct Poisson solve initialized by a specific chronological extrapolation of the mesh potential. This result explains, for the first time, the origin of the accelerated convergence observed in the present and previous MaZe-based electrostatic formulation, recasting what had previously been an empirical observation as a controlled feature of the formalism. Because the underlying extrapolation is independent of the constrained-dynamics formulation itself, this mechanism also provides a recipe for constructing (or rederiving) effective initial guesses for conventional iterative real-space Poisson solvers. We assess P3MaZe by comparing the computed electrostatic potential, structural, and dynamical observables for molten NaCl and SPC/Fw water against reference Poisson MaZe, P3M and Ewald calculations, and by conducting a detailed analysis of computational performance and solver convergence.

## Theory

Consider a system of *N*_*p*_ point particles with coordinates {***r***_β_}, masses {*m*_β_}, and charges {*Q*_β_}. The corresponding microscopic charge density is

1 $$\rho \left(r\right)=\overset{N_{p}}{\underset{\beta =1}{\sum }}Q_{\beta }\delta \left(r-r_{\beta }\right)$$

Under periodic boundary conditions, the electrostatic energy includes the interaction of each charge with all of its periodic replicas and is given by

2 $$U_{c}=\frac{1}{2}\underset{n\in Z^{3}}{\sum }\overset{N_{p}}{\underset{\alpha =1}{\sum }}{\overset{N_{p}}{\underset{\beta =1}{\sum }}}^{\prime }\frac{Q_{\alpha }Q_{\beta }}{\left|r_{\alpha \beta }+n_{cell}\right|}$$

where ***r***_αβ_ = ***r***_α_ – ***r***_β_, ***n***_cell_ = *n*_1_ ***a***_1_ + *n*_2_ ***a***_2_ + *n*_3_ ***a***_3_ is a lattice vector of the simulation cell, and the prime excludes the singular self-interaction α = β for ***n***_cell_ = **0**. The lattice sum is conditionally convergent and is defined according to the usual Ewald convention.^1^ Following the standard particle–mesh formulation,^13^ the periodic Coulomb interaction is decomposed into a rapidly decaying short-range contribution and a smooth long-range contribution by introducing a screening charge density associated with each particle. The total screening density is

3 $$\rho^{s}\left(r\right)=\overset{N_{p}}{\underset{\beta =1}{\sum }}\rho_{\beta }^{s}\left(r;\left\{r_{\beta }\right\}\right)$$

In the present work we adopt Gaussian screening functions,

4 $$\rho_{\beta }^{s}\left(r;\left\{r_{\beta }\right\}\right)=\frac{Q_{\beta }}{{\left(2\pi \sigma^{2}\right)}^{3/2}}\exp\left[-\frac{|r-r_{\beta }{|}^{2}}{2\sigma^{2}}\right]$$

consistent with the formulation of Beckers.^25^ The electrostatic energy is written, exactly, as

5 $$U_{c}=U_{SR}+U_{LR}+U_{self}$$

With our choice for the screening density, the short-range contribution, given by the difference between the Coulomb interaction of the point charges and that of the corresponding screening densities, is

6 $$U_{SR}=\frac{1}{2}\underset{n\in Z^{3}}{\sum }\overset{N_{p}}{\underset{\alpha =1}{\sum }}{\overset{N_{p}}{\underset{\beta =1}{\sum }}}^{\prime }\left[\frac{Q_{\alpha }Q_{\beta }}{\left|r_{\alpha \beta }+n_{cell}\right|}-\int drdr^{\prime }\frac{\rho_{\alpha }^{s}\left(r;\left\{r_{\alpha }\right\}\right)\rho_{\beta }^{s}\left(r^{\prime };\left\{r_{\beta }\right\}\right)}{\left|r-r^{\prime }+n_{cell}\right|}\right]≃\frac{1}{2}\overset{N_{p}}{\underset{\alpha =1}{\sum }}\overset{r_{\alpha \beta }<R_{c}}{\underset{\beta \neq \alpha }{\sum }}\frac{Q_{\alpha }Q_{\beta }}{r_{\alpha \beta }}\left[1-\mathrm{erf}\left(\frac{r_{\alpha \beta }}{\sqrt{2}\sigma }\right)\right]$$

In the second line, the rapidly decaying short-range contribution is evaluated using the minimum-image convention and truncated at the real-space cutoff *R*_*c*_. For the cubic cells considered here, *R*_*c*_ ≤ *L*/2. The magnitude of the contribution in the expression above, i.e., the short-range tail beyond *R*_*c*_, is controlled by the choice of *R*_*c*_ and σ. The term

7 $$\begin{array}{ll} U_{self} & =-\frac{1}{2}\overset{N_{p}}{\underset{\alpha =1}{\sum }}\int drdr^{\prime }\frac{\rho_{\alpha }^{s}\left(r;\left\{r_{\alpha }\right\}\right)\rho_{\alpha }^{s}\left(r^{\prime };\left\{r_{\alpha }\right\}\right)}{\left|r-r^{\prime }\right|}\\ & =-\frac{1}{\sqrt{2\pi }\sigma }\overset{N_{p}}{\underset{\alpha =1}{\sum }}Q_{\alpha }^{2} \end{array}$$

removes the self-interaction of each screening density.

The smooth long-range interaction is

8 $$U_{LR}=\frac{1}{2}\underset{n\in Z^{3}}{\sum }\overset{N_{p}}{\underset{\alpha =1}{\sum }}\overset{N_{p}}{\underset{\beta =1}{\sum }}\int drdr^{\prime }\frac{\rho_{\alpha }^{s}\left(r;\left\{r_{\alpha }\right\}\right)\rho_{\beta }^{s}\left(r^{\prime };\left\{r_{\beta }\right\}\right)}{\left|r-r^{\prime }+n_{cell}\right|}$$

which retains the full periodic long-range electrostatic contribution. Rather than evaluating eq 8 directly, particle–mesh methods determine the corresponding electrostatic potential by solving the Poisson equation

9 $$\nabla^{2}\phi_{LR}\left(r\right)=-4\pi \rho^{s}\left(r\right)$$

which, after discretization on a uniform grid of spacing *h*, becomes

10 $$M\phi_{LR}=-4\pi \frac{q^{s}}{h}$$

where ***M***/*h* ^2^ is the discrete Laplacian and ***q*** ^*s*^/*h* ^3^ is the vector of assigned smooth charges. Under periodic boundary conditions, the discrete Laplacian ***M*** has a constant null mode, so the electrostatic potential is defined only up to an additive constant. We fix this freedom by imposing the gauge ⟨**ϕ**_LR_⟩ = 0 after each field update. Within the constrained-dynamics formulation described below, this gauge condition can equivalently be interpreted as an additional linear constraint selecting a unique representative of the mesh potential.

Up to this point, the formulation of the problem follows the standard particle–mesh formulation. The remaining numerical task is the solution of the discrete Poisson equation at every simulation step. Conventional particle–mesh methods perform this solve using either FFT-based reciprocal-space techniques or iterative real-space solvers.

The novel idea that P3MaZe brings in is to reformulate the long-range particle–mesh problem as a constrained dynamical system. Instead of solving the discrete Poisson equation (eq 10 ) at every step along a MD trajectory, the values of the long-range electrostatic potential on the mesh are promoted to auxiliary dynamical variables. The discrete Poisson equation is then enforced as a holonomic constraint within the MaZe formalism, following the approach previously developed for Poisson MaZe.^35^

To this end, the components of the discretized long-range electrostatic potential, **ϕ**_LR_, are assigned a finite auxiliary inertia μ (whose vanishing limit will be considered below), leading to the extended Lagrangian

$$L_{P3MaZe}=\frac{1}{2}\overset{N_{p}}{\underset{\beta =1}{\sum }}m_{\beta }{ṙ}_{\beta }^{T}{ṙ}_{\beta }+\frac{1}{2}\mu {\mathrm{\phi ̇}}_{LR}^{T}{\mathrm{\phi ̇}}_{LR}-\mathrm{U̅}\left(\left\{r_{\beta }\right\}\right)-U_{SR}\left(\left\{r_{\beta }\right\}\right)-U_{LR}\left(\phi_{LR};\left\{r_{\beta }\right\}\right)-U_{self}-\lambda^{T}\sigma_{LR}\left(\phi_{LR};\left\{r_{\beta }\right\}\right)$$ 11

where $\mathrm{U̅}\left(\left\{r_{\beta }\right\}\right)$ contains all nonelectrostatic interactions and **λ** denotes the vector of Lagrange multipliers associated with the constraints. The discretized long-range electrostatic energy is

12 $$U_{LR}\left(\phi_{LR};\left\{r_{\beta }\right\}\right)=\frac{h}{8\pi }\phi_{LR}^{T}M\phi_{LR}+\phi_{LR}^{T}q^{s}$$

and the holonomic constraints are defined as the stationarity conditions of the long-range energy,

13 $$\sigma_{LR}\left(\phi_{LR};\left\{r_{\beta }\right\}\right)=\nabla_{\phi_{LR}^{T}}U_{LR}\left(\phi_{LR};\left\{r_{\beta }\right\}\right)=\frac{h}{4\pi }M\phi_{LR}+q^{s}\left(\left\{r_{\beta }\right\}\right)=0$$

Equation 13 is the discrete Poisson equation (eq 10) expressed as a holonomic constraint. Consequently, the constraint manifold coincides with the set of mesh potentials satisfying the discrete Poisson equation. Furthermore, when the constraint is satisfied,

14 $$U_{LR}=\frac{1}{2}\phi_{LR}^{T}q^{s}$$

which is the standard discrete expression for the electrostatic energy of the smooth charge distribution.^36^

The evolution equations for the extended system follow from the Euler–Lagrange equations. As in the general MaZe formalism,^29,35^ the physical dynamics are recovered in the limit μ → 0. In this limit, the Lagrange multipliers scale linearly with μ, ensuring that the acceleration of the auxiliary variables remains finite. As shown in Section 1 of the Supporting Information, the resulting equations of motion are

15 $$\begin{array}{ll} m_{\alpha }{\mathrm{r̈}}_{\alpha } & =-\nabla_{r_{\alpha }}U_{SR}\left(\left\{r_{\beta }\right\}\right)-\nabla_{r_{\alpha }}U_{LR}\left(\phi ;\left\{r_{\beta }\right\}\right)-\nabla_{r_{\alpha }}\mathrm{U̅}\left(\left\{r_{\beta }\right\}\right),\\ {\mathrm{\phi ̈}}_{LR} & =-M\eta \end{array}$$

where $\eta =\lim_{\mu \to 0}\frac{h}{4\pi }\frac{\lambda }{\mu }$ is the rescaled vector of Lagrange multipliers.

In practice, the physical degrees of freedom are propagated using the velocity–Verlet algorithm, while the auxiliary variables are integrated using the Verlet scheme. At each time step, the rescaled Lagrange multipliers are determined by enforcing the constraint $\sigma_{LR}\left(\phi_{LR};\left\{r_{\beta }\right\}\right)=0$ via an adapted SHAKE algorithm.^37^ Thus, the dynamics evolve directly on the constraint manifold defined by eq 13, ensuring that the mesh potential remains consistent with the smooth charge density throughout the simulation.

### P3MaZe and P3M: Constrained propagation and temporal extrapolation

We now show that, for the linear (in the auxiliary variables) Poisson constraint considered here, the solution of the P3MaZe constraint problem and the direct real-space Poisson solve are algebraically equivalent, and that this equivalence gives the constrained dynamics a previously unrecognized interpretation as a chronological predictor for the mesh potential obtained via standard P3M solvers. As mentioned above, in a conventional real-space P3M approach, the long-range potential is obtained by directly solving the discretized Poisson equation at each MD step using a chosen initial guess. In P3MaZe, the same solution is reached instead by propagating the auxiliary mesh potential and correcting it so as to satisfy the Poisson constraint. At time step *k* + 1, the full update of the mesh potential in P3MaZe is given by a standard Verlet step

16 $$\phi_{LR}^{k+1}=\phi_{LR,p}^{k+1}-\frac{4\pi }{h}y^{k+1}$$

where $\phi_{LR,p}^{k+1}$ denotes the provisional potential predicted by the Verlet propagation, $\phi_{LR,p}^{k+1}=2\phi_{LR}^{k}-\phi_{LR}^{k-1}$ , and ***y*** ^*k*+1^ is the SHAKE correction that enforces the constraint. As detailed in the Supporting Information, the latter is related to the rescaled Lagrange multipliers through

17 $$y^{k+1}=\frac{h}{4\pi }\Delta t^{2}M\eta^{k+1}$$

The constraint dynamics then focuses on obtaining the unknown quantity ***y*** ^*k*+1^ rather than directly the potential. These corrections are determined via the SHAKE condition

18 $$\sigma_{LR}\left(\phi_{LR}^{k+1};\left\{r_{\beta }^{k+1}\right\}\right)=0$$

by substituting the Verlet prediction for the potential in the argument

19 $$\sigma_{LR}\left(\phi_{LR,p}^{k+1}-\frac{4\pi }{h}y^{k+1};\left\{r_{\beta }^{k+1}\right\}\right)=0$$

The equation above can be trivially rewritten as

20 $$My^{k+1}=\frac{h}{4\pi }M\phi_{LR,p}^{k+1}+q^{s,k+1}\equiv \sigma_{LR,p}^{k+1}$$

where $\sigma_{LR,p}^{k+1}$ is the value of the constraint computed at provisional potential. This argument shows that the discrete Poisson equation for the potential at step *k* + 1, eq 18, and the SHAKE solution for ***y*** ^*k*+1^ are algebraically equivalent formulations of the linear system for the long-range electrostatic potential. The first is typically solved iteratively after input of the initial guess for the potential. The second is also solved iteratively, possibly - as done later in this work - with the same solver used for standard Poisson, but can be conveniently interpreted as a predictor-corrector scheme in which the predictor is the provisional potential and the correction with the SHAKE step.

An interesting consequence of the discussion above pertains to the initialization of the iterative solve. In P3MaZe, the initial guess is specified for the correction variable ***y*** not directly for the potential. We denote by $y_{0}^{\left(m\right)}$ a predictor constructed from *m* previous converged corrections, with $y_{0}^{\left(0\right)}=0$ . Through eq 16, each choice of $y_{0}^{\left(m\right)}$ determines a corresponding initial estimate of the mesh potential that can be fed to the direct solver. We denote by $\phi_{LR,0}^{\left(m\right)}$ the initial mesh-potential estimate corresponding to the correction predictor $y_{0}^{\left(m\right)}$ .

For $y_{0}^{\left(0\right)}=0$ , the initial potential is simply the provisional Verlet prediction,

21 $$\phi_{LR,0}^{\left(0\right)}=\phi_{LR,p}^{k+1}=2\phi_{LR}^{k}-\phi_{LR}^{k-1}$$

A more informative initialization is obtained by retaining the converged correction from the preceding time step. For the standard P3MaZe choice $y_{0}^{\left(1\right)}=y^{k}$ , the update relation at the preceding time step gives

22 $$\phi_{LR,0}^{\left(1\right)}=\phi_{LR,p}^{k+1}-\frac{4\pi }{h}y^{k}=3\phi_{LR}^{k}-3\phi_{LR}^{k-1}+\phi_{LR}^{k-2}$$

Thus, reusing the previous converged correction is exactly equivalent, for the fixed linear operator considered here, to a quadratic chronological extrapolation of the converged mesh potential.

More generally, for the uniform time step used here, higher-order predictors can be constructed from the *m* most recent converged corrections as

23 y0(m)=∑j=0m−1(−1)j(mj+1)yk−j,⁡m≥1

Using eq 16 and the corresponding relations at the preceding time steps, this gives

24 ϕLR,0(m)=∑j=0m+1(−1)j(m+2j+1)ϕLRk−j

Thus, the constrained propagation provides an explicit correspondence between predictors for the correction variable and chronological predictors for the mesh potential. Equation 24 is not strictly dependent on its dynamical origin and can therefore be employed to initialize different iterative solvers for real space electrostatics.

In general, temporal extrapolation of previous solutions, including Lagrange polynomial predictors, is a well-established strategy for accelerating sequences of iterative linear solves.^38^ In P3MaZe, the extrapolation mechanism arises naturally from the constrained propagation of the auxiliary mesh potential, providing a direct dynamical basis for the higher-order predictors introduced above. In previous work, Beckers et al.^25^ noted that the converged electrostatic potential from the previous ionic configuration can provide an effective initial guess for the subsequent solve. However, attempts to improve on this via a Taylor expansion in time of the potential did not prove fruitful for the systems they considered and was not pursued further. Connected strategies have also been developed in molecular simulation methods involving auxiliary or self-consistent variables but not necessarily transferred to electrostatics in classical MD. For example, Köstler et al. used previous Hartree potentials together with - only - second order temporal extrapolation to initialize successive Poisson solves in first-principles Car–Parrinello MD.^39^ For the simulation of classical polarizable models, Kolafa introduced a predictor–corrector scheme based on the history of self-consistent polarization variables.^40^ The Kolafa scheme has been adapted also to first principle MD in the so-called second-generation Car–Parrinello dynamics.^41^ However, the predictor–corrector construction of Kolafa requires to tune system dependent parameters and is not fully time-reversible. In contrast, because the P3MaZe predictor-corrector scheme is embedded in the constrained dynamics of the system, it automatically inherits convenient properties such as symplecticity and time-reversibility.

### P3MaZe and Poisson MaZe: Charge Assignment and Smoothing

An important distinction between Poisson MaZe and P3MaZe concerns the source term of the mesh Poisson equation. In Poisson MaZe, the full particle charge density is assigned directly to the grid and acts as the source of the discretized Poisson problem. In P3MaZe, by contrast, the source term is the smooth screening charge density arising from the particle–mesh decomposition, as in conventional real-space P3M methods.^25,26^ Consequently, P3MaZe inherits the systematic accuracy framework of particle–mesh electrostatics. In the present implementation, particle charges are first assigned to the mesh using cubic B-splines. The resulting charge density is subsequently smoothed over a finite stencil to obtain the mesh density ***q*** ^*s*^ entering eq 13. This two-step construction provides the discrete particle–mesh representation of the continuous Gaussian screening density introduced above. The B-spline assignment is part of the mesh discretization, rather than an additional physical smoothing, and its effect is consistently accounted for by using the same B-spline weights when interpolating the mesh field back to the particles before computing the force. The resulting particle-level force therefore represents the long-range force generated by the screened Gaussian charge density within the adopted mesh discretization, as discussed in the paragraph on electrostatic forces. Because the source term is smooth, the corresponding mesh potential is also smoother than in Poisson MaZe, with reduced high-frequency components of the mesh potential. This in turn reduces the mesh resolution required to achieve a given target accuracy and therefore the number of necessary auxiliary variables. This additional smoothing operation introduces an extra particle–mesh cost that is absent in Poisson MaZe. As shown in the Results, however, this overhead is more than compensated by the reduced cost of the solver, leading to an overall improvement in computational efficiency.

### P3MaZe and Poisson MaZe: Electrostatic Energy

In Poisson MaZe, the electrostatic potential is obtained from the full discretized Poisson problem. Consequently, a direct evaluation of the mesh energy contains a grid self-energy associated with the discrete representation of each particle charge.^13,35^ This contribution depends on both the charge-assignment scheme and the mesh resolution, making its removal numerically delicate. For this reason, Poisson MaZe evaluates changes in the electrostatic energy from the mechanical work $U_{c}^{work}$ performed by the electrostatic forces along the trajectory,

25 $$U_{c}^{work}\left(t\right)-U_{c}^{work}\left(0\right)=-\overset{N_{p}}{\underset{\alpha =1}{\sum }}\int_{r_{\alpha }^{0}}^{r_{\alpha }\left(t\right)}F_{\alpha }\cdot dr_{\alpha }$$

where $U_{c}^{work}\left(0\right)$ fixes the arbitrary energy reference. In P3MaZe, the singular Coulomb interaction is treated analytically through *U*_SR_, while the mesh represents only the smoothed long-range contribution *U*_LR_. Consequently, the grid self-energy associated with discretized point charges is absent, and the only remaining self-contribution is the analytical Gaussian screening correction given by eq 7. The smoothing procedure provides a well-behaved explicit estimate of the long-range energy *U*_LR_ (eq 14).

At finite mesh resolution, the work-derived energy is more consistent with the computed forces than the explicit mesh energy and is therefore used in the NVE analysis. However, by the work–energy theorem, changes in kinetic energy are compensated, up to time-integration and work-quadrature errors, by opposite changes in the energy reconstructed from those forces. The conservation of the resulting total energy is therefore primarily a consistency test between force propagation and work integration, rather than an independent validation of the dynamics. Independent stability tests are instead provided by kinetic energy or temperature drift and by their dependence on the time step and solver tolerance. The comparison between the two energy definitions and the NVE energy tests are reported in Section 2 of the Supporting Information.

### Electrostatic Forces

The electrostatic force follows the same decomposition as the energy,

26 $$F_{\alpha }=F_{\alpha }^{SR}+F_{\alpha }^{LR}$$

where the short-range contribution is evaluated analytically. For the Gaussian screening function (eq 4),

27 $$F_{\alpha }^{SR}=\overset{r_{\alpha \beta }<R_{c}}{\underset{\beta \neq \alpha }{\sum }}Q_{\alpha }Q_{\beta }\left[\frac{\mathrm{erfc}\left(r_{\alpha \beta }/\sqrt{2}\sigma \right)}{r_{\alpha \beta }^{3}}+\sqrt{\frac{2}{\pi }}\frac{\exp\left[-r_{\alpha \beta }^{2}/\left(2\sigma^{2}\right)\right]}{\sigma r_{\alpha \beta }^{2}}\right]r_{\alpha \beta }$$

where ***r***_αβ_ = ***r***_α_ – ***r***_β_ (*r*_αβ_ = | ***r***_αβ_|), and the sum is restricted to particle pairs within the real-space cutoff *R*_*c*_.

The long-range contribution is obtained from the constrained mesh potential using the same interpolation procedure employed in Poisson MaZe. For example, the (*x*)-component is

28 $$F_{\alpha ,x}^{LR}=-Q_{\alpha }\underset{m}{\sum }W\left(r_{\alpha }-r_{m}\right)D_{x}\phi_{LR,m}$$

where *D*_*x*_ denotes the finite-difference approximation to the mesh gradient and *W* is the particle–mesh interpolation function. Greek subscripts label particles, whereas Latin subscripts denote mesh points. In the present implementation, *W* is a cubic B-spline, identical to the charge-assignment function. Using the same interpolation function for charge assignment and force reconstruction ensures consistency between the two operations, as in Poisson MaZe.

The P3MaZe force construction preserves the momentum-conserving properties of both components of the particle–mesh decomposition. The short-range contribution is pairwise and antisymmetric and therefore conserves total momentum exactly (up to floating-point roundoff). As in Poisson MaZe, the long-range contribution conserves momentum on the mesh provided that charge assignment and force interpolation employ the same weight function and the Laplacian is discretized using central finite differences.^13^ In the present implementation, both conditions are satisfied by using cubic B-splines together with the standard seven-point finite-difference stencil. Higher-order Hermitian discretizations^26^ could be adopted straightforwardly if greater mesh accuracy were required.

Taken together, these differences show that P3MaZe combines the systematic accuracy and analytical structure of particle–mesh electrostatics with the constrained-dynamics formulation and efficient iterative correction strategy of Poisson MaZe. Momentum conservation tests and further discussion on the topic can be found in Section 2 of the Supporting Information.

## Results

### Molten Salt

#### System and Simulation Protocol

As a first validation of the method, we consider molten NaCl, which provides a direct comparison with our previous Poisson MaZe formulation.^35^ Since P3MaZe differs from Poisson MaZe only in the treatment of the long-range electrostatic problem, this system allows us to isolate the effect of introducing the particle–mesh decomposition while keeping the underlying constrained-dynamics framework unchanged. In a later section, and in the Supporting Information, we also report results on the accuracy of the electrostatic potential computed with P3MaZe compared to a standard P3M solver.

The simulated system consists of 250 ions (125 Na^+^ and 125 Cl^–^) in a cubic simulation cell of side *L* = 20.64 Å, corresponding to a density of 1.3793 g·cm^–3^. Periodic boundary conditions are applied in all directions. Nonelectrostatic interactions are described by the Born–Huggins–Meyer potential with Tosi–Fumi parameters, following the model adopted in previous simulations of molten NaCl.^35,42,43^

Initial configurations were generated from a bcc lattice with Maxwell–Boltzmann velocities at *T* = 1550 K. After equilibration in the canonical ensemble using Langevin dynamics integrated with the OVRVO splitting scheme,^44^ production trajectories were generated in the microcanonical ensemble by setting the friction coefficient to zero, thereby recovering the velocity–Verlet limit. The equilibration stage lasted 2.5 ps with a time step of 0.25 fs. For P3MaZe, the electrostatic parameters were chosen according to the convergence analysis reported in Section 3 of the Supporting Information. The Gaussian screening width was set to σ = 1.39 Å, corresponding to an Ewald splitting parameter β = 0.5087 Å^–1^, the real-space cutoff was *R*_*c*_ = 4.5 Å, and the mesh consisted of 60^3^ grid points (*h* = 0.344 Å). The smooth charge density was generated using the diffusion-based smoothing procedure of Beckers and co-workers,^25,26^ whose computational cost scales linearly with the number of mesh points. The number of diffusion steps *N*_diff_ is selected so that the variance of the discrete diffusion kernel reproduces the target Gaussian width σ: *N*_diff_ = round [(σ/*h*)^2^/(2*D*_*g*_)], where *h* is the mesh spacing and *D*_*g*_ = 1/6.2 is the dimensionless diffusion coefficient.^26^ For comparison, Poisson MaZe calculations employed the optimized setup of ref 35 using a 120^3^ mesh (*h* = 0.172 Å). In both methods, the constraint equations were solved at every molecular dynamics step to a tolerance of 10^–7^.

#### Structural and Dynamical Validation

The purpose of the following analysis is to establish that replacing the full-grid Poisson formulation of Poisson MaZe by the particle–mesh decomposition of P3MaZe preserves both equilibrium and dynamical properties. We therefore compare structural, single-particle transport, and collective transport observables obtained with the two methods against available literature data.

The equilibrium liquid structure is first assessed through the partial radial distribution functions. Figure 1 compares the Na–Cl, Na–Na and Cl–Cl distributions obtained from 25 ps microcanonical trajectories with those reported by Galamba et al.^42^ The P3MaZe and Poisson MaZe curves are indistinguishable within graphical resolution for all ionic pairs, demonstrating that introducing the particle–mesh decomposition does not modify the equilibrium structure of the liquid. Dynamical observables provide a more stringent validation because they accumulate the effect of the electrostatic forces over extended trajectories. We first examine the velocity autocorrelation function (VACF), from which the self-diffusion coefficients are obtained as

29 $$D_{a}=\frac{1}{3N_{a}}\underset{\alpha \in a}{\sum }\int_{0}^{\infty }dt\left\langle v_{\alpha }^{a}\left(t\right)\cdot v_{\alpha }^{a}\left(0\right)\right\rangle$$

where *a* denotes the ionic species and the average is taken over the *N*_*a*_ particles of that species and over time origins.

**Figure 1:**
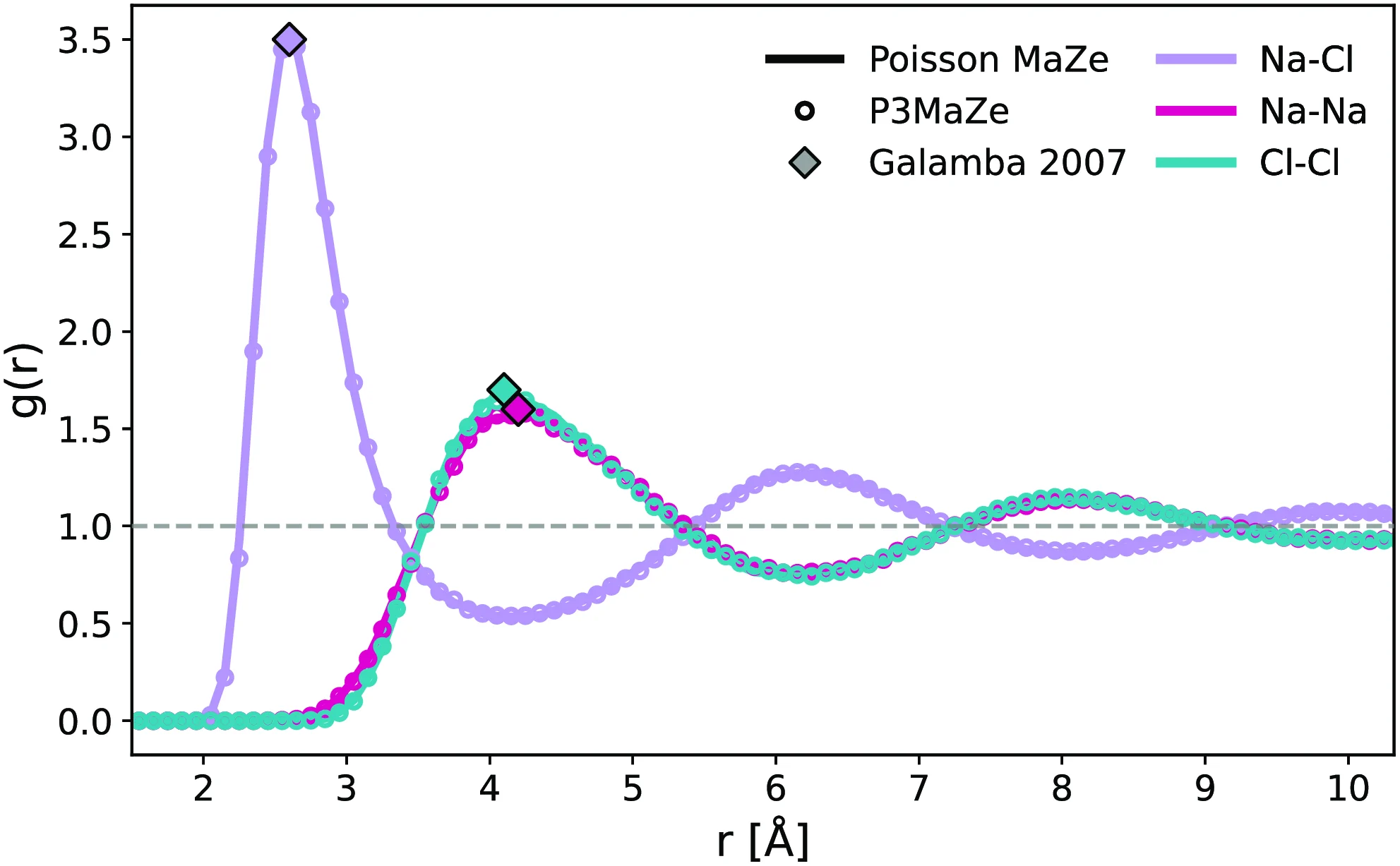
Partial radial distribution functions of molten NaCl obtained with Poisson MaZe and P3MaZe. The Na–Cl, Na–Na and Cl–Cl distributions are compared with the reference peak positions reported by Galamba et al.^42^ The two methods are in quantitative agreement, indicating that the particle–mesh decomposition preserves the equilibrium liquid structure.

Diffusion coefficients were estimated from ensembles of ten independent 15 ps microcanonical trajectories for each electrostatic method. Statistical uncertainties correspond to the standard error of the replica averages. Figure 2a compares the normalized VACFs together with the cumulative diffusion coefficients. Agreement between Poisson MaZe and P3MaZe is observed over the entire correlation time, leading to statistically indistinguishable diffusion coefficients: *D*_Na_ = (0.161 ± 0.004) × 10^–3^ cm^2^·s^–1^ and *D*_Cl_ = (0.144 ± 0.004) × 10^–3^ cm^2^·s^–1^ for Poisson MaZe, compared with *D*_Na_ = (0.159 ± 0.004) × 10^–3^ cm^2^·s^–1^ and *D*_Cl_ = (0.146 ± 0.004) × 10^–3^ cm^2^·s^–1^ for P3MaZe. Both methods also agree with the reference values of Galamba et al.^42^

**Figure 2:**
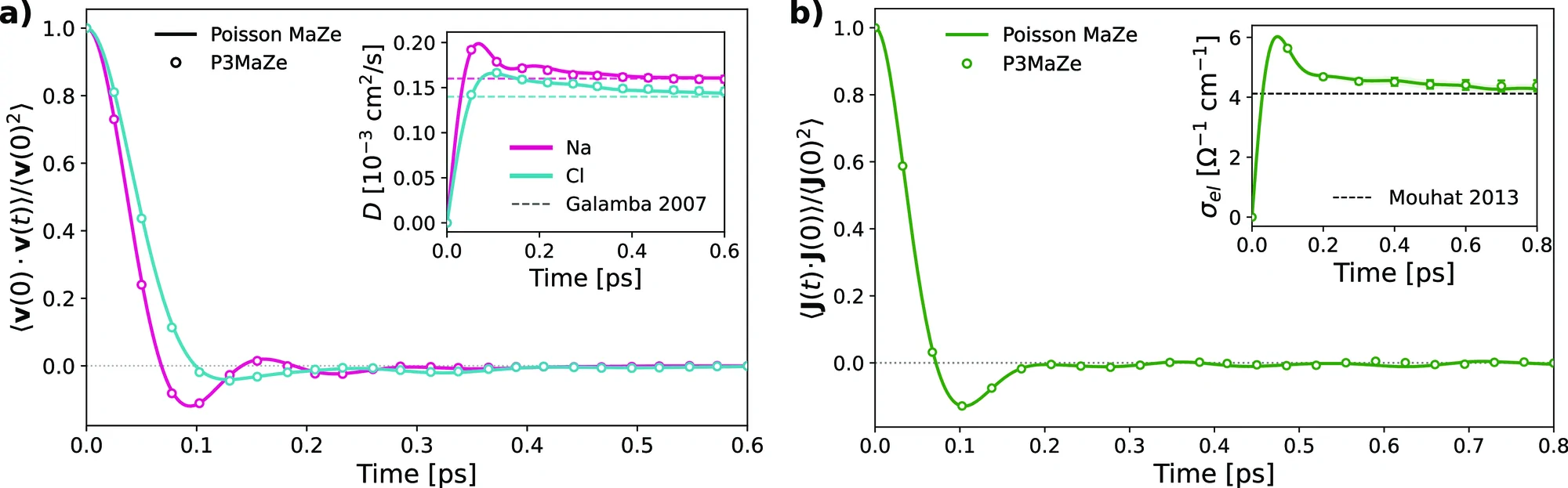
Comparison of single-particle and collective dynamical observables for molten NaCl obtained with Poisson MaZe and P3MaZe. (a) VACF of Na^+^ and Cl^–^ ions together with the corresponding cumulative Green–Kubo integrals used to determine the self-diffusion coefficients. (b) CACF and cumulative Green–Kubo integrals yielding the electrical conductivity. Shaded regions indicate the standard error estimated from independent trajectories. The two methods yield statistically indistinguishable translational dynamics and finite-time molecular charge responses, demonstrating that introducing the particle–mesh decomposition into the MaZe framework preserves both single-particle and collective dynamical properties.

As a more demanding test, we next consider the current autocorrelation function (CACF), with the current computed as $J\left(t\right)=\overset{N_{p}}{\underset{\alpha =1}{\sum }}Q_{\alpha }v_{\alpha }\left(t\right)$ . This correlation probes collective charge transport and is therefore particularly sensitive to the treatment of long-range electrostatic interactions.^25^ The corresponding electrical conductivity was obtained from

30 $$\sigma_{el}\left(t\right)=\frac{1}{3Vk_{B}T}\int_{0}^{t}\left\langle J\left(0\right)\cdot J\left(t^{\prime }\right)\right\rangle dt^{\prime }$$

using ensembles of 20 independent 40 ps trajectories. In the equation above *V* is the volume of the system. Figure 2b shows that the current autocorrelation functions and cumulative conductivities obtained with Poisson MaZe and P3MaZe are statistically indistinguishable. The resulting conductivities, σ_el_ = 4.29 ± 0.15 Ω^–1^·cm^–1^ for Poisson MaZe and σ_el_ = 4.39 ± 0.18 Ω^–1^·cm^–1^ for P3MaZe, are mutually consistent and remain close to the reference value reported by Mouhat et al.^43^

These structural and dynamical results demonstrate that introducing the particle–mesh decomposition into the MaZe framework leaves both equilibrium and transport properties unchanged within statistical uncertainty. The P3M decomposition therefore preserves the physical accuracy of the original constrained formulation while substantially reducing the computational cost of the long-range electrostatic treatment, as quantified in the performance analysis shown later in this work.

### SPC/Fw Model

Having established that P3MaZe reproduces the original Poisson MaZe formulation for a strongly ionic liquid, we consider a substantially more demanding benchmark based on the flexible SPC/Fw water model introduced by Wu, Tepper, and Voth.^45^ Unlike molten salts, liquid water is not dominated by strong ionic correlations or high screening effects. Long-range electrostatic interactions instead play a central role in determining the hydrogen bond network, molecular reorientation, and transport properties, making water a particularly sensitive test of the accuracy of the electrostatic treatment.

SPC/Fw is a flexible three-site model in which the interaction sites coincide with the atomic nuclei. Each water molecule is described by two O–H bond lengths and one H–O–H bond angle (all represented as harmonic interactions), allowing the intramolecular degrees of freedom to couple continuously to the electrostatic environment. The parameters of the model were taken from ref 45 and are summarized in the Supporting Information. As in the original SPC/Fw model, Lennard–Jones interactions act only on the oxygen site.

#### System and Simulation Protocol

In contrast with the molten-salt benchmark, where the comparison isolates the effect of introducing the particle–mesh decomposition into the MaZe framework, the SPC/Fw system assesses whether P3MaZe reproduces the structural and dynamical properties obtained with established particle–mesh electrostatic methods. The primary comparison is performed against the P3M implementation in LAMMPS^46^ through time-resolved structural and dynamical observables. As an additional consistency check, integrated observables are also compared with Ewald summation and the original Poisson MaZe formulation (Table 1).

**Table 1:** Integrated Structural and Dynamical Observables for SPC/Fw Water Obtained with P3M, P3MaZe, Ewald, and Poisson MaZe at 298.15 K^a^

observable	P3M	P3MaZe	Ewald	Poisson MaZe	ref value
*D* [10^–5^ cm^2^/s]	2.29 ± 0.04	2.33 ± 0.04	2.30 ± 0.04	2.56 ± 0.05	2.32 ± 0.05
σ (80–100 ps) [Ω^–1^/cm]	–0.022 ± 0.031	0.014 ± 0.023	–0.018 ± 0.022	–	–
τ_1_ (dipole) [ps]	4.88 ± 0.16	4.83 ± 0.14	4.88 ± 0.10	4.58 ± 0.10	4.70
τ_2_ (dipole) [ps]	1.60 ± 0.04	1.58 ± 0.04	1.62 ± 0.04	1.51 ± 0.04	1.72
τ_1_ (H–H) [ps]	4.70 ± 0.12	4.44 ± 0.10	4.49 ± 0.11	4.28 ± 0.10	3.90
τ_2_ (H–H) [ps]	2.00 ± 0.04	1.98 ± 0.05	2.05 ± 0.05	1.95 ± 0.05	2.01
τ_1_ (O–H) [ps]	4.79 ± 0.12	4.60 ± 0.12	4.52 ± 0.10	4.38 ± 0.10	4.17
τ_2_ (O–H) [ps]	1.80 ± 0.04	1.81 ± 0.04	1.81 ± 0.04	1.75 ± 0.04	1.86

a P3MaZe is statistically consistent with the established particle–mesh methods across all observables, while the comparison with Poisson MaZe highlights the improvement obtained by introducing the particle–mesh decomposition. Reference values are taken from ref 45 unless stated otherwise.

The P3M and Ewald calculations were performed with LAMMPS, whereas P3MaZe and Poisson MaZe employed our in-house implementation. All four methods were applied to identical molecular configurations and simulation conditions. The simulated system consists of 216 water molecules in a cubic periodic box of side *L* = 18.552 Å, corresponding to a density of 1.012 g·cm^–3^, at *T* = 298.15 K. After a 1 ns equilibration in the canonical ensemble using a time step of 0.5 fs, production data were collected from 40 distinct 150 ps microcanonical trajectories. Initial configurations for the production runs were extracted every 250 fs from the equilibrated trajectory and used for all four electrostatic methods. For the charge-current analysis, we performed an additional set of ten independent 200 ps microcanonical trajectories using P3M, Ewald summation, and P3MaZe. These calculations were designed to resolve the short-time molecular charge response and examine its long-time limit. The microscopic charge current was recorded every five MD steps, corresponding to a sampling interval of 2.5 fs, and the finite-time integrated response was evaluated up to 100 ps. Poisson MaZe was not included in this additional long-time charge-current analysis because its SPC/Fw results exhibit a substantially stronger sensitivity to spatial resolution, as independently demonstrated by the diffusion and rotational observables. The purpose of these additional simulations is to establish the expected long-time limit of the charge response and verify that P3MaZe agrees with the established P3M and Ewald references. We note that, while the number of molecules and the real-space cutoff were chosen consistently with ref 45, those simulations were performed in the (N,P,T) ensemble, whereas the present production calculations are carried out at fixed volume in (N,V,E); differences with the reported reference values may therefore arise from the different thermodynamic protocols.

For the LAMMPS calculations, the real-space cutoff was 9.0 Å, while the Ewald splitting parameter was determined automatically from the requested force accuracy of 10^–7^, consistent with the original SPC/Fw parametrization.^45^ The corresponding splitting parameter was then used to define the Gaussian width of the P3MaZe decomposition (σ = 1.87 Å). The real-space cutoff for P3MaZe was selected using the same convergence procedure described for molten NaCl in Section 3 of the Supporting Information, yielding *R*_*c*_ = 6.0 Å. A mesh of 40^3^ grid points was found sufficient to converge all structural and dynamical observables discussed below. For Poisson MaZe, the electrostatic potential was represented on a uniform 180^3^ mesh.

#### Observables

As mentioned above, the SPC/Fw benchmark assesses whether P3MaZe reproduces the structural and dynamical properties obtained with established particle–mesh electrostatic methods. We therefore compare time-resolved observables against the LAMMPS P3M implementation and summarize the corresponding integrated quantities for all four electrostatic methods (P3M, P3MaZe, Ewald, and Poisson MaZe) in Table 1.

We first examine the equilibrium liquid structure through the oxygen–oxygen, oxygen–hydrogen, and hydrogen–hydrogen radial distribution functions. Figure 3 compares the P3M and P3MaZe results together with the reference peak positions reported by Wu et al.^45^ The two methods are statistically indistinguishable over the full range of intra- and intermolecular separations, demonstrating that the P3MaZe particle–mesh decomposition preserves the equilibrium structure of liquid water.

**Figure 3:**
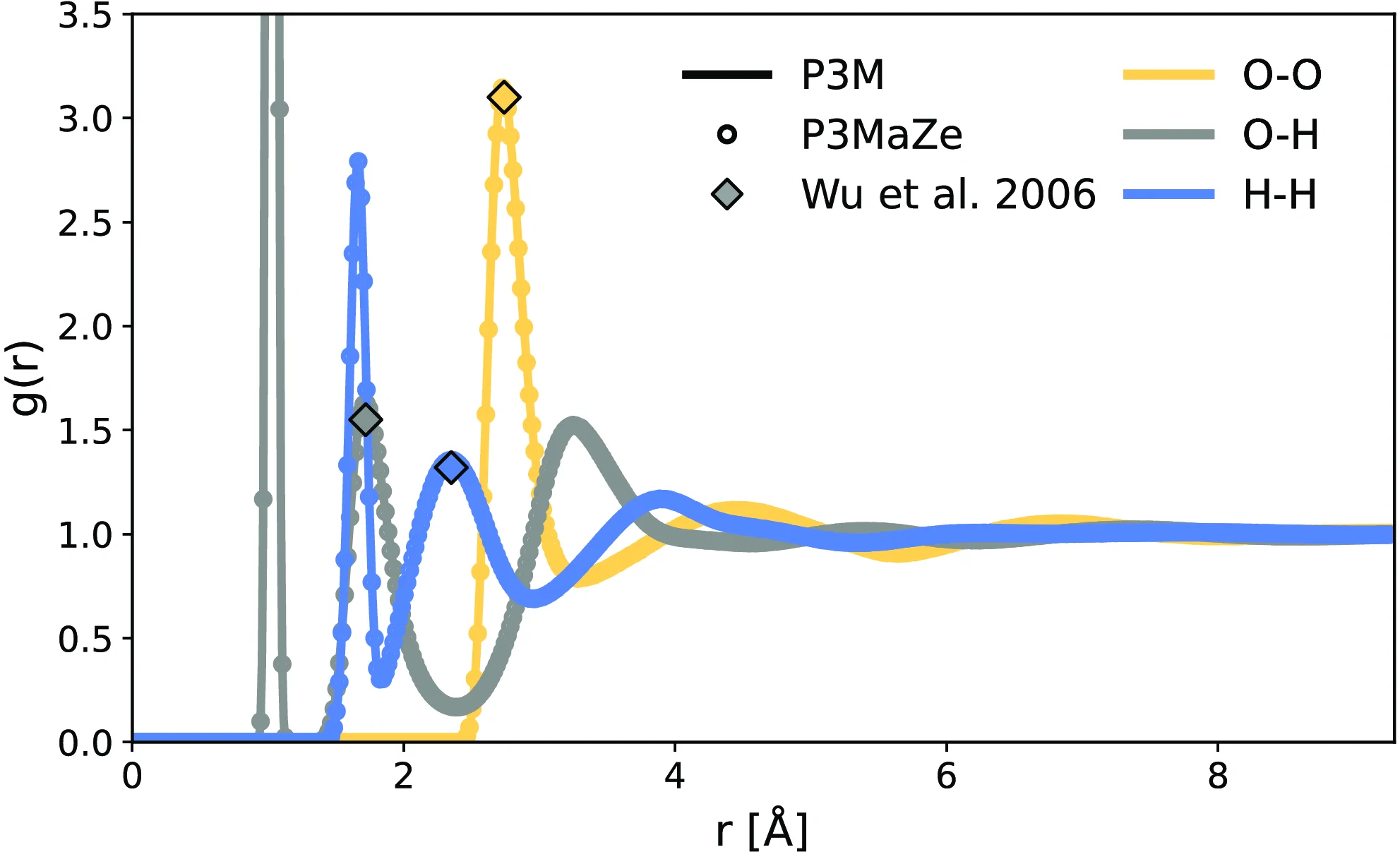
Comparison of oxygen–oxygen, oxygen–hydrogen and hydrogen–hydrogen radial distribution functions for SPC/Fw water obtained with P3M and P3MaZe. Diamond symbols indicate the reference peak positions reported by Wu et al.^45^ The agreement between the curves demonstrates that P3MaZe preserves the equilibrium structure of liquid water.

As for the dynamical observables, we first consider the molecular VACF and the corresponding cumulative Green–Kubo integral for the self-diffusion coefficient. Figure 4a shows that the normalized VACFs obtained with P3M and P3MaZe overlap over the entire correlation time, yielding nearly identical running diffusion coefficients and statistically indistinguishable plateau values. The corresponding numerical estimates are reported in Table 1, confirming that P3MaZe reproduces the translational dynamics of liquid water with the same accuracy as the standard P3M reference.

**Figure 4:**
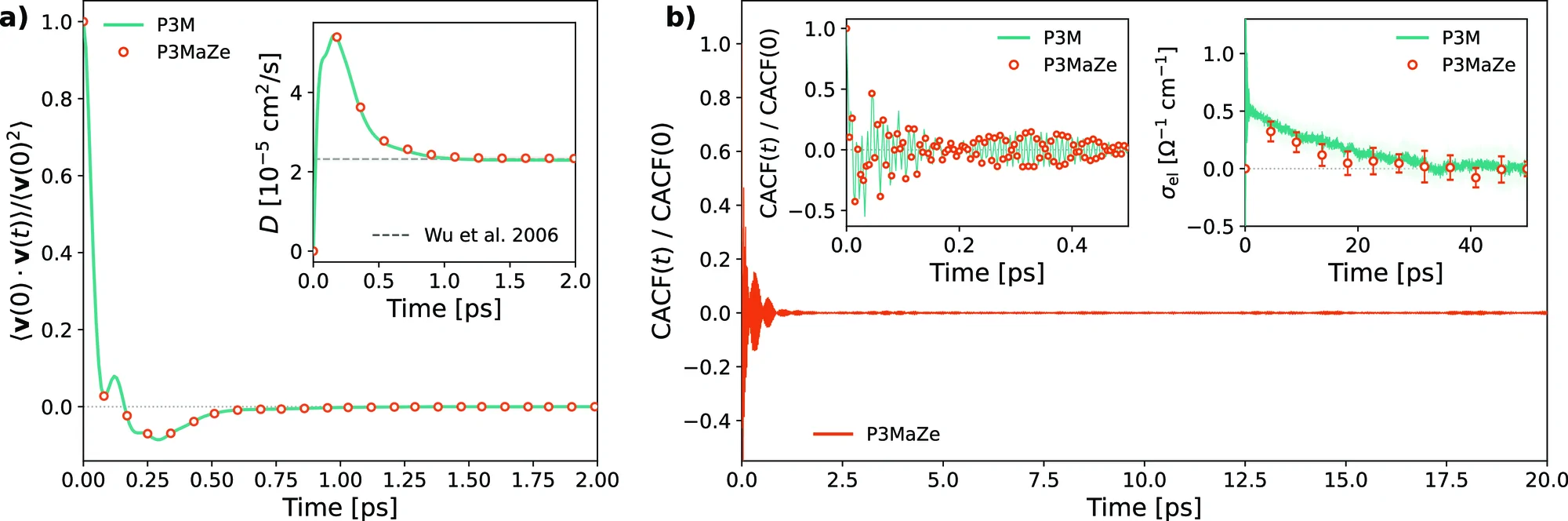
Comparison of single-particle and collective dynamical observables for SPC/Fw water obtained with P3M and P3MaZe. (a) Normalized molecular VACF together with the cumulative Green–Kubo integrals used to determine the self-diffusion coefficient. (b) Normalized CACF; the main panel shows the first 20 ps, while the left inset resolves the first 0.5 ps and the right inset reports the finite-time integrated charge-current response. P3MaZe results are shown as symbols with error bars, whereas P3M is represented by a solid line with a shaded region indicating the standard error of the mean across the independent replicas. The two methods produce statistically indistinguishable correlation functions and transport coefficients, demonstrating that both preserve both translational dynamics and collective electrostatic response.

We next examine the collective molecular charge response through the CACF and its time integral, shown in Figure 4b. Due to the molecular vibrations and librations, the CACF exhibits a nonzero oscillatory signal at short times and progressively decays toward zero, as highlighted in the left inset. The corresponding running time integral is shown in the right inset. P3MaZe and P3M results for these quantities agree within statistical uncertainty. For a precise calculation of the residual long-time contribution, we average the running integral of the current autocorrelation function separately for each replica over the interval 80–100 ps and estimate the standard error across the ten replica averages. The values obtained with P3M, Ewald, and P3MaZe, reported in Table 1, are all statistically compatible with zero, as expected for a neutral system with no net charge transport. These results provide a sensitive probe of the long-range electrostatic correlations, in the same spirit as previous assessments of Coulomb solvers.^25^ As a final dynamical validation, we consider rotational relaxation. Rotational correlation functions probe molecular reorientation and therefore complement the translational and collective transport observables discussed above. Figure 5 reports the first- and second-order rotational correlation functions of the molecular dipole together with their cumulative relaxation times. The corresponding definitions are

31 $$C_{1}\left(t\right)=\left\langle P_{1}\left[u\left(t\right)\cdot u\left(0\right)\right]\right\rangle =\left\langle u\left(t\right)\cdot u\left(0\right)\right\rangle$$

and

32 $$C_{2}\left(t\right)=\left\langle P_{2}\left[u\left(t\right)\cdot u\left(0\right)\right]\right\rangle =\left\langle \frac{1}{2}\left(3{\left[u\left(t\right)\cdot u\left(0\right)\right]}^{2}-1\right)\right\rangle$$

where **u** is the molecular unit vector associated with the chosen axis. For the results reported below, ***u*** is chosen along the molecular dipole direction. The main panel reports the correlation functions, while the inset shows the cumulative time integrals whose plateau values define the relaxation times τ_1_ and τ_2_. Excellent agreement between P3M and P3MaZe is observed for both the correlation functions and the integrated relaxation times. The corresponding results for the H–H and O–H molecular axes are reported in Section 5 of the Supporting Information, while all integrated relaxation times are summarized in Table 1.

**Figure 5:**
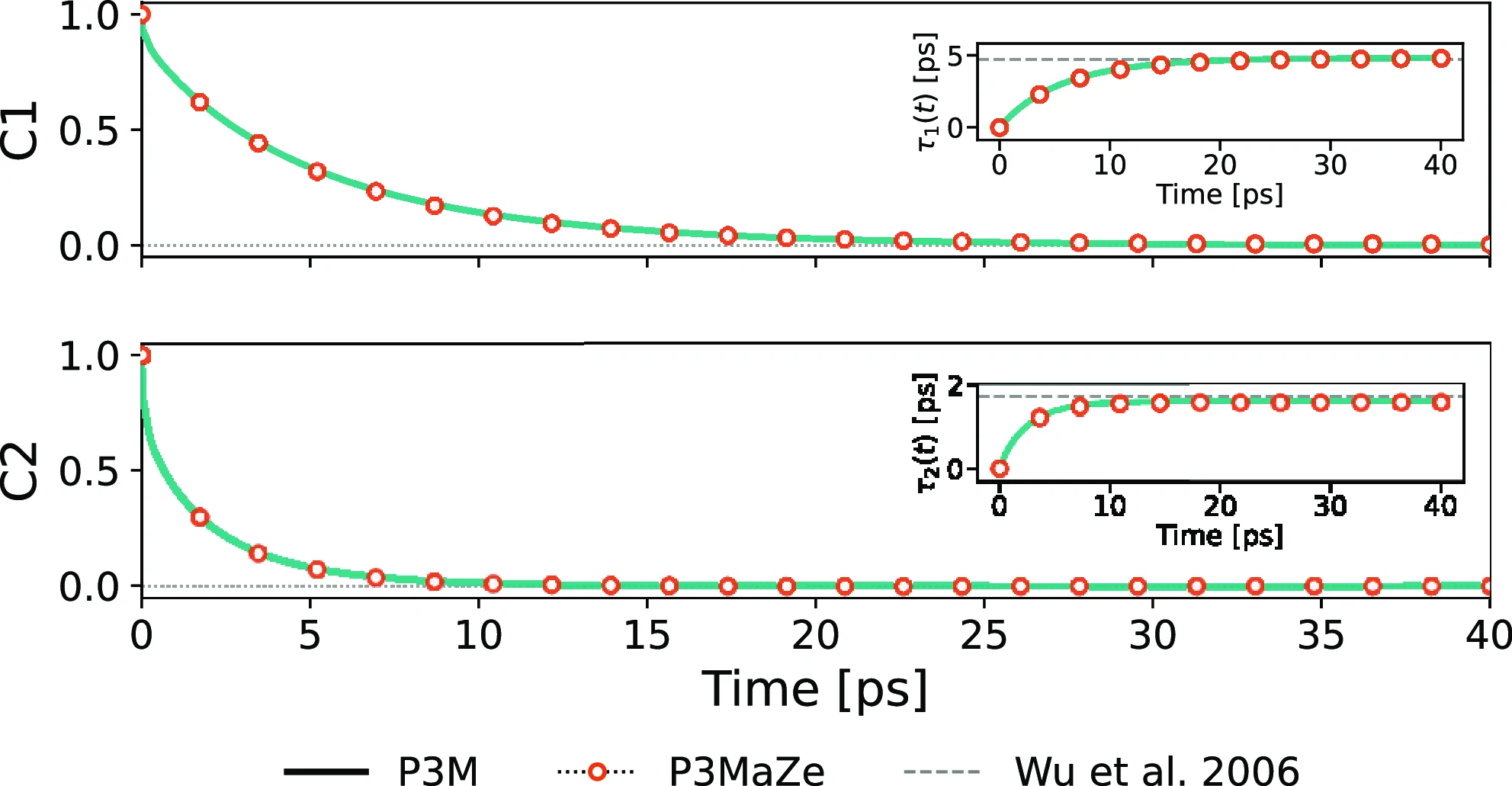
Comparison of dipole rotational relaxation in SPC/Fw water obtained with P3M and P3MaZe. The main panel reports the normalized first- and second-order rotational correlation functions, while the inset shows the corresponding cumulative relaxation times. Solid lines denote P3M and symbols denote P3MaZe. The close agreement between the two methods demonstrates that P3MaZe accurately reproduces the orientational dynamics of liquid water.

The integrated observables for all four methods are collected in Table 1. P3M, P3MaZe, and Ewald yield mutually consistent estimates across all quantities, in satisfactory agreement with the reference values of Wu et al.^45^ Poisson MaZe, by contrast, systematically overestimates the self-diffusion coefficient and shows larger deviations in the rotational relaxation times, even at the high spatial resolution of *N* = 180 grid points per direction (*h* = 0.103 Å). This shows the sensitivity of Poisson MaZe to grid resolution in systems with weak ionic screening: unlike molten salts, liquid water presents a more demanding long-range electrostatic environment in which the full Coulomb grid problem converges more slowly with mesh refinement.

Taken together, these results indicate that P3MaZe preserves not only the structural properties of SPC/Fw water but also both its single-particle and collective dynamical behavior.

### Performance and Scaling

The objective of this part of the paper is to quantify the computational consequences of the P3MaZe formulation and, in particular, the role of the initial guess generated by auxiliary-field propagation. We compare P3MaZe with both Poisson MaZe and an in-house real-space P3M implementation. The latter serves as a numerical illustration of the equivalence of the two methods and of their numerical solution, provided that a coherent initialization is adopted for the solver. A direct pointwise comparison of the corresponding electrostatic potentials, including their dependence on rigid translations relative to the mesh, is reported in Section 6 of the Supporting Information to further substantiate this equivalence.

In Figure 6 we show the average maximum-norm residual as a function of the number of iterations for NaCl (left panel) and SPC/Fw water (right panel) computed with Poisson MaZe (purple curves) and P3MaZe (different colors identify different initializers for the iterative multigrid solver). The maximum-norm residual is defined, for all methods, as

33 $${res}_{\nu }=\underset{n}{\max}\left|\left\{{\left(\frac{h}{4\pi }M\phi_{LR}^{\nu }+q^{s}\right)}_{n}\right\}\right|$$

where ν is the index of the iteration and *n* runs over the mesh.

**Figure 6:**
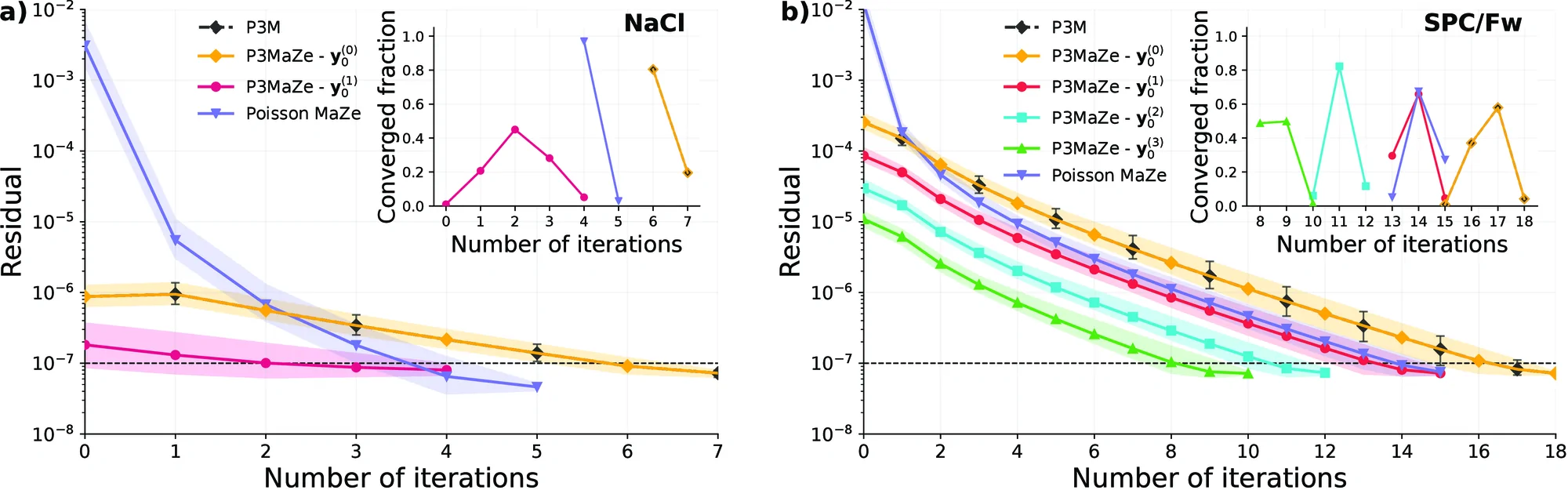
Residual convergence of the long-range electrostatic solver for (a) molten NaCl and (b) SPC/Fw water. Lines denote the mean residual decay. The minimum-to-maximum spread over sampled MD steps and restart configurations is represented by shaded regions for P3MaZe and Poisson MaZe, and by error bars for P3M. With the zero-correction predictor, P3MaZe reproduces the convergence of the corresponding real-space P3M solver, whereas predictors constructed from the correction history progressively reduce the number of iterations. The inset in each panel reports the iteration-count distribution over the sampled data.

The data were obtained from the same restart configurations used for the independent dynamic replicas discussed in the previous subsections. For each restart and each method, short trajectories of 100 MD steps were propagated and the residual was recorded after each multigrid iteration during the field update. The initial steps of each trajectory were discarded because they are affected by the initialization of the field solver and converge more slowly than the subsequent plateau regime (see also Figure 8). The plotted curves therefore report the residual decay averaged over the remaining steps and restart configurations. The minimum-to-maximum spread across the analyzed samples is shown by shaded regions for P3MaZe and Poisson MaZe and by error bars for P3M.

In both panels of Figure 6, the yellow curve reports results for P3MaZe initialized with $y_{0}^{\left(0\right)}=0$ . The black symbols superimposed on this curve are obtained via a direct solution of the discretized Poisson equation with the same multigrid solver initialized according to eq 21. The excellent agreement of the data illustrates P3M/P3MaZe equivalence. For the molten salt, Figure 6a, we also show that, when the value of the SHAKE correction at the previous time step, $y_{0}^{\left(1\right)}$ , is instead used as the initial guess, P3MaZe reaches the prescribed tolerance in one or two iterations, and always within one to four iterations over the sampled steps. By contrast, Poisson MaZe requires four to five iterations. These results are more clearly visible in the inset of the figure that reports the iteration count distribution for the sampled data. This acceleration reflects the combined effect of the MaZe correction mechanism and the smoother long-range source term entering the P3MaZe constraint.

The SPC/Fw system, Figure 6b, provides a more demanding test of the iterative solver. All methods require more iterations than those in molten NaCl, reflecting the weaker electrostatic screening of liquid water and the larger integration time step, both of which reduce the correlation between successive mesh updates. As in molten NaCl, P3MaZe with $y_{0}^{\left(0\right)}=0$ closely reproduces the convergence of the direct P3M solve, consistent with the algebraic equivalence discussed above. This system also allows us to better assess different initializations of the iterative SHAKE solution.

In addition to the reference choice $y_{0}^{\left(0\right)}=0$ , we consider the cases *m* = 1, 2, and 3 of the correction predictor defined in eq 23. As established in eq 24, these choices correspond to quadratic, cubic, and quartic chronological extrapolations of the converged mesh potential, respectively. Together with the linear Verlet prediction associated with ***y***_0_^(0)^, they provide a systematic hierarchy of increasingly accurate temporal initializations. Using ***y***_0_^(1)^, P3MaZe converges in approximately 13–14 iterations, already below the baseline of ***y***_0_^(0)^/P3M of 16–17 iterations. Poisson MaZe requires 17–19 iterations and therefore offers no convergence advantage over P3M for this weakly screened molecular system. Higher-order predictors substantially improve the P3MaZe convergence: ***y***_0_^(2)^ reduces the iteration count to approximately 11, while ***y***_0_^(3)^ further reduces it to 8–9, nearly halving the number of iterations relative to the direct real-space P3M solve. The systematic reduction with predictor order demonstrates how the solver acceleration is controlled by the quality of the temporal initialization.

This dependence can be further examined considering the behavior of the initial residual as a function of the time step Δ*t* employed in the MD trajectory. For any time-dependent quantity with a bounded (*m* + 2)­th derivative over the extrapolation interval, the standard error formula for polynomial interpolation predicts that a degree-(*m* + 1) Lagrange extrapolation has an error of order Δ*t* ^*m*+2^; see ref 47, Theorem 2.1.4.1. Accordingly, the residual is expected to decrease with increasing predictor order according to the corresponding power-law scaling in Δ*t*. Figure 7 shows that the theoretical trend is very closely matched by our simulations. (Data reported for the case of SPC/Fw water.) The result can be attributed to the smooth variation of the potential with time driven in turn by the smooth variations of the mesh charge that follow the continuous changes in the ions positions along the trajectory and is preserved by our mesh assignment and smoothing operations.

**Figure 7:**
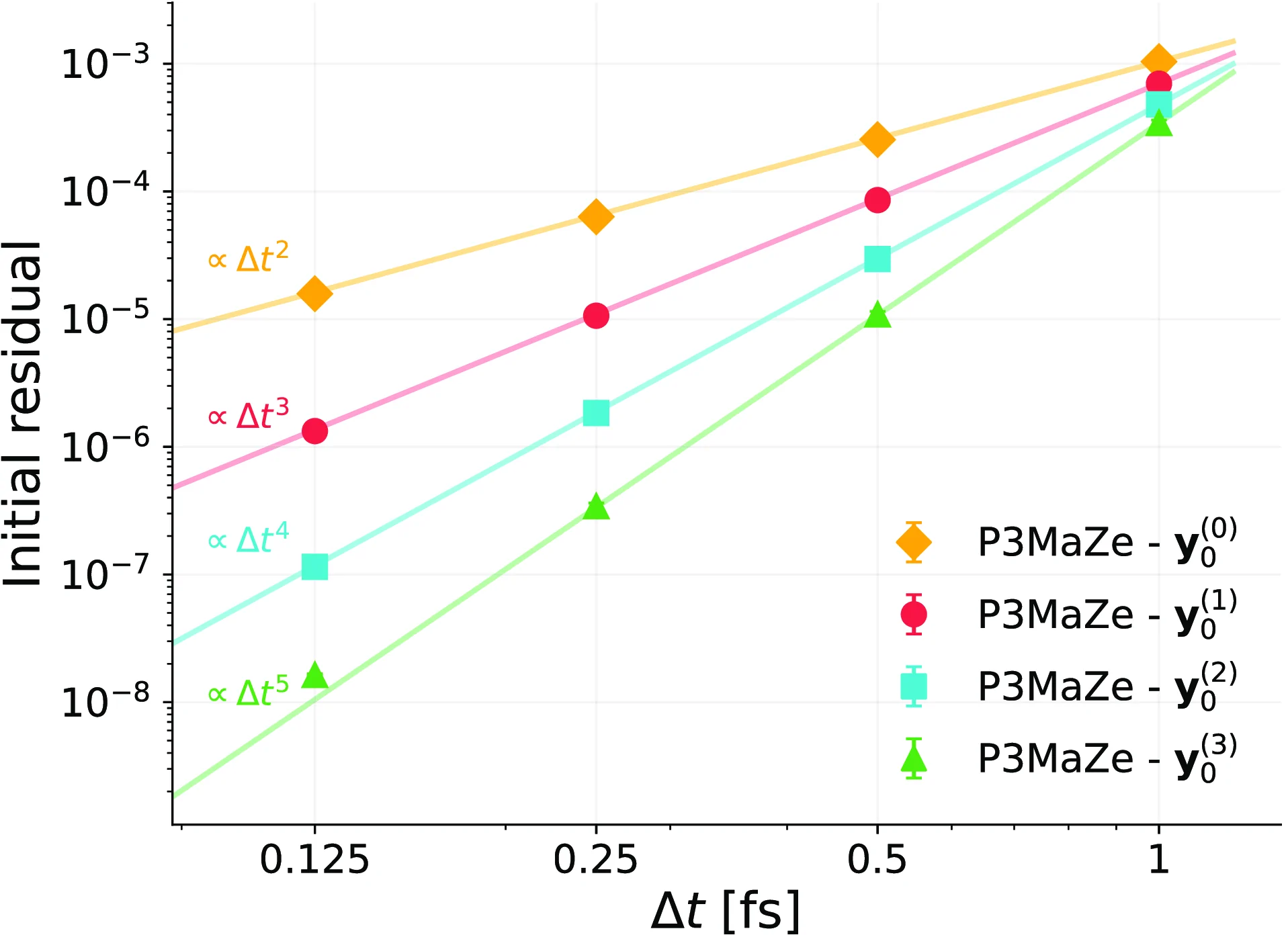
Initial residual of the long-range electrostatic solve for SPC/Fw water as a function of the integration time step Δ*t*. Results are shown for the correction predictors $y_{0}^{\left(m\right)}$ , with *m* = 0, 1, 2, 3. The reference lines indicate the expected asymptotic scalings Δ*t* ^*m*+2^.

The reduction in the initial residual shown in Figure 7 translates into a stable, lower number of multigrid iterations during MD, as shown in Figure 8. In both systems, the first steps exhibit a short initialization transient before reaching a steady-state plateau. Within this regime, the iteration count decreases systematically with predictor order with the largest reduction observed for SPC/Fw water.

**Figure 8:**
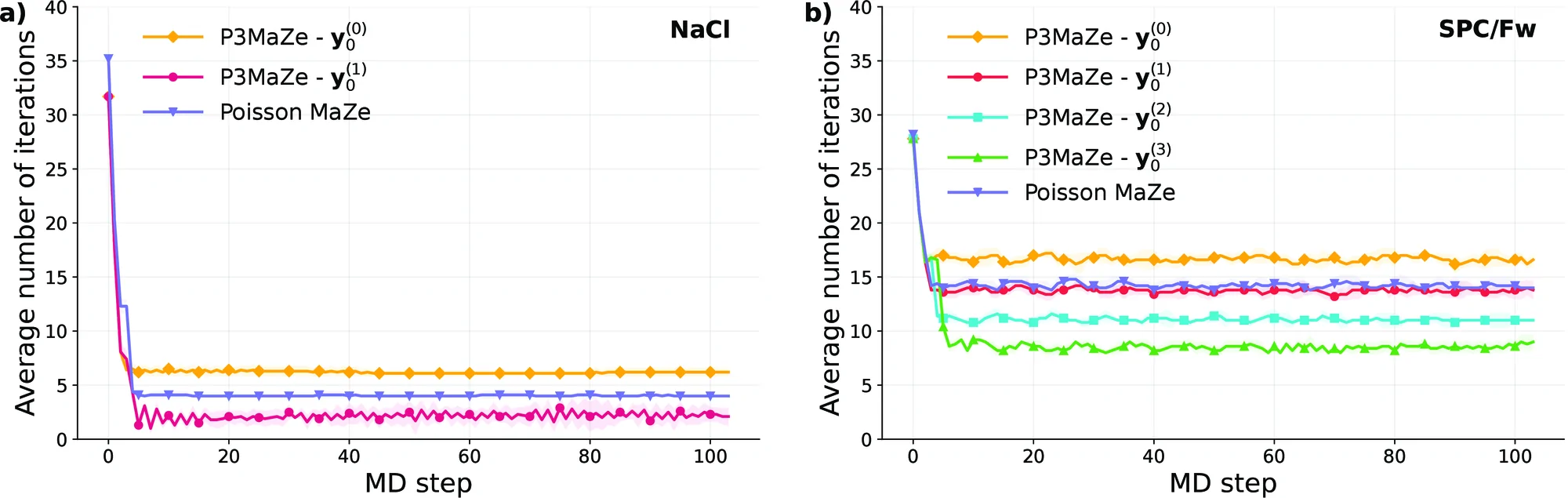
Average number of multigrid iterations per MD step for (a) molten NaCl and (b) SPC/Fw water. The faster residual convergence observed in Figure 6 translates into a sustained reduction in the number of multigrid iterations during MD, and higher-order predictors progressively improve the P3MaZe convergence in the more demanding SPC/Fw system.

We conclude our analysis by demonstrating the linear scaling of the algorithm with the overall number of degrees of freedom. Figure 9 reports the electrostatic CPU time per MD step as a function of system size on logarithmic axes for molten NaCl (panel a) and SPC/Fw water (panel b). The mesh resolution was scaled to preserve the mesh spacing *h* of the corresponding production simulations as *N*_*p*_ was increased. For each system, all simulations were performed at the same density. Linear fits in the log–log representation confirm the expected O(N) scaling of the real-space formulation, while the vertical offsets between the curves quantify the reduction in computational cost achieved by the different initializations. We note that, in addition to the properties of the multigrid solver, linear scaling is obtained due to the sparse nature of the discretized Laplacian with PBC that only has the same seven nonzero entries per row.^48^

**Figure 9:**
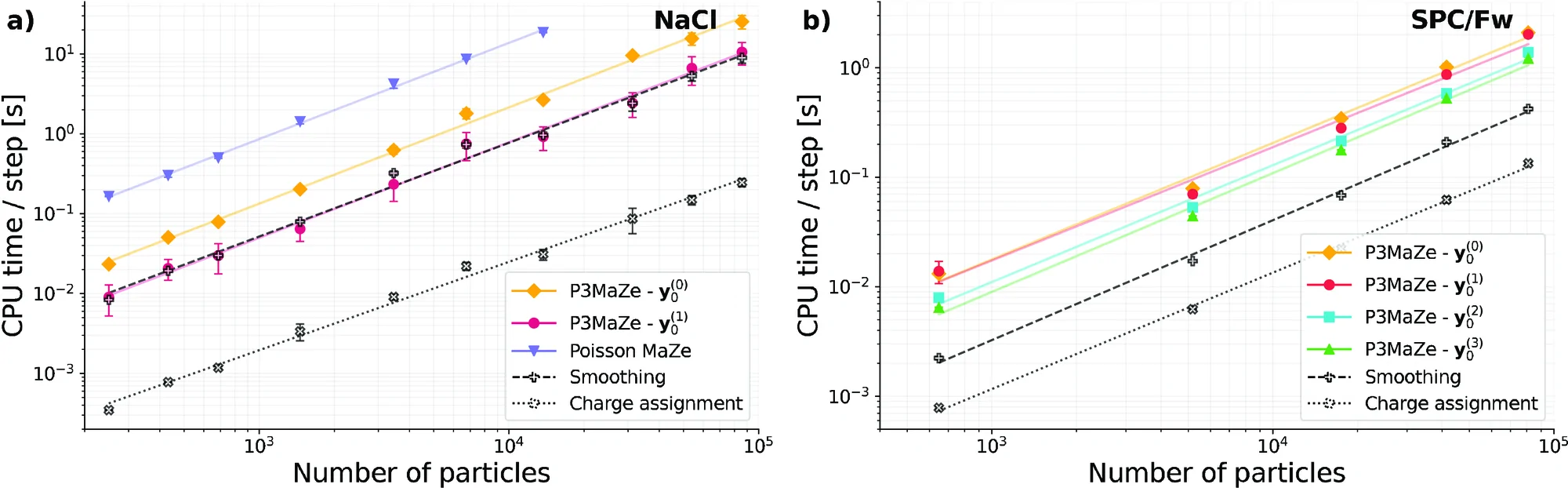
Electrostatic CPU time per MD step as a function of system size shown on logarithmic axes. Linear fits confirm the expected O(N) scaling of all real-space formulations, while the vertical separation between the curves quantifies the reduction in computational cost obtained with P3MaZe. Shared charge-assignment and smoothing costs are shown separately. (a) Molten NaCl. (b) SPC/Fw water.

This enables us to perform matrix vector multiplication with O(N) scaling and very low memory requirements. In the present implementation, ***M*** is not explicitly assembled or stored. Its action is evaluated directly through the seven-point finite difference stencil applied to the vector. Furthermore, the storage required by the different initialization of the iterative cycle is respectively one, two, and three previous ***y*** vectors, each containing N elements; thus, the predictor does not alter the O(N) memory requirements.

For molten NaCl, Figure 9a, all methods exhibit approximately unit slopes, confirming the expected linear scaling of the underlying multigrid solver. Poisson MaZe is the most expensive method. Considering the mesh-solver contribution alone, linear fits of the untransformed CPU times as functions of system size yield a timing coefficient approximately 19 times larger than that of P3MaZe, reflecting the cost of solving the full Coulomb problem on the fine 120^3^ reference grid. When charge assignment and smoothing are included, the ratio between the total electrostatic timing coefficients decreases to approximately 9, since smoothing accounts for about half of the total P3MaZe cost. The corresponding solver-only and total electrostatic fit coefficients are reported in Table S3 of the Supporting Information.

Identical results would be found for a real-space P3M adopting the chronological initialization of the potential. As the improved initialization reduces the long-range solver cost, the smoothing step becomes the dominant contribution to the P3MaZe electrostatic time, accounting for approximately 50% of the total cost across the system sizes considered. The same linear scaling is observed for SPC/Fw water, Figure 9b. Here, Poisson MaZe is omitted because the fine mesh required for the full Coulomb grid problem makes it considerably expensive at the larger system sizes. For this system, smoothing rises to 15–30% of the computational overhead in the P3MaZe variants as the solver contribution decreases with improved initial guess.

These results show that the particle–mesh decomposition and the MaZe propagation play complementary roles. The decomposition reduces the size and smooths the source of the long-range mesh problem, while the temporal information naturally available in the MaZe dynamics provides increasingly accurate initial guesses for the iterative solve. The resulting method preserves the systematic accuracy controls of particle–mesh electrostatics while reducing the cost of the long-range solver. The timing analysis also identifies the next optimization target: once the multigrid correction is accelerated, particle–mesh preprocessing, and in particular the smoothing step, becomes the main remaining bottleneck.

## Conclusions

We have introduced P3MaZe, a real-space particle–mesh electrostatic method that combines the standard short-range/long-range decomposition of P3M with the Mass-Zero constrained-dynamics framework. In this formulation, the smooth long-range electrostatic potential is treated as a zero-inertia auxiliary field whose evolution is constrained by the discretized Poisson equation. As a result, the long-range mesh problem is solved through the constrained evolution of the auxiliary variables. This formulation combines the complementary strengths of particle–mesh electrostatics and constrained dynamics. By retaining the standard P3M decomposition, P3MaZe preserves the systematic accuracy controls associated with the real-space cutoff, the Ewald splitting, the mesh spacing, and the charge-assignment procedure. At the same time, casting the long-range solve as a holonomic constraint on zero-inertia auxiliary variables endows P3MaZe with the structural properties of constrained MaZe dynamics: the equations of motion are symplectic and time-reversible, the auxiliary field remains adiabatically separated from the physical degrees of freedom in the μ → 0 limit, and the resulting trajectories exhibit the expected long-term stability. Within this structurally robust setting, the auxiliary-field propagation additionally provides effective initial guesses for the iterative long-range solve.

The numerical benchmarks demonstrate that this reformulation efficiently preserves the physical accuracy of particle–mesh electrostatics. For molten NaCl, P3MaZe reproduces the structural and dynamical properties obtained with the original Poisson MaZe formulation and with literature reference data. For flexible SPC/Fw water, a substantially more demanding electrostatic benchmark, P3MaZe yields structural, translational, collective, and rotational dynamical observables that are statistically indistinguishable from those obtained with LAMMPS P3M and Ewald summation.

The solver analysis clarifies the origin of the low iterative cost observed in P3MaZe, and in doing so establishes a new result for the MaZe framework itself. For the linear, time-independent Poisson operator considered here, we show that the MaZe correction problem is algebraically equivalent to the corresponding direct Poisson solve, with the two connected by an explicit, closed-form chronological extrapolation of the mesh potential. This analysis identifies, for the first time, the precise mechanism responsible for the accelerated convergence previously observed in MaZe-based electrostatic solvers, turning what had been an empirical feature of the formalism into a controlled and predictable one. Because the extrapolation formula is independent of the constrained-dynamics construction itself, our results indicate that MaZe dynamics identifies effective initializations for conventional iterative solvers. In both benchmark systems, exploiting this temporal information reduces the number of multigrid iterations substantially, and higher-order predictors constructed along the same lines provide additional gains in the more demanding SPC/Fw simulations. The timing analysis confirms that P3MaZe preserves the expected linear scaling of real-space particle–mesh electrostatics while reducing the prefactor associated with the long-range mesh solve. As the cost of the constrained long-range solve decreases, charge assignment and smoothing become an increasingly important fraction of the total electrostatic cost, identifying the particle–mesh preprocessing stage as the next target for algorithmic optimization.

The results presented in this paper provide a flexible foundation for further algorithmic developments for P3MaZe and equivalent P3M solvers, including improved particle–mesh preprocessing, more effective predictor strategies, and implementations targeting highly parallel many-core and GPU architectures.

## Supplementary Material



## Data Availability

The data and software underlying this study are available through Materials Cloud at https://archive.materialscloud.org/records/x415p-e7q15. The archive includes the source code, simulation input files, processed data, and analysis scripts required to reproduce the results reported in the manuscript and Supporting Information.

## Notes

Derivation of the P3MaZe equations of motion and numerical integration scheme; NVE stability and conservation tests; convergence tests and parameter tuning for P3MaZe; SPC/Fw force-field parameters; additional rotational relaxation functions for the H–H and O–H molecular axes; a direct pointwise comparison of the P3M and P3MaZe electrostatic potentials, including their translational dependence relative to the mesh; and numerical details of the performance scaling analysis (PDF)
