# Supplementary material for: P3MaZe: a Mass-Zero Constrained-Dynamics Formulation of Particle–Mesh Electrostatics
Source: J Chem Theory Comput. 2026 Oct 26;22(21):11442–55. doi: 10.1021/acs.jctc.6c01315 (PMC13757764; doi:10.1021/acs.jctc.6c01315)
Supplement: Supplementary file 1 [file ct6c01315_si_001.pdf]

Supporting Information: P3MaZe: A Mass-Zero constrained-dynamics formulation of particle–mesh electrostatics

Federica Troni,[†] Violette Gontran,[†] Davide Grassano,[†] and Sara Bonella[*,†]

†*Centre Européen de Calcul Atomique et Moléculaire (CECAM), Ecole Polytechnique Fédérale de Lausanne, 1015 Lausanne, Switzerland*

‡*National Centre for Computational Design and Discovery of Novel Materials (MARVEL), Ecole Polytechnique Fédérale de Lausanne, CH-1015 Lausanne, Switzerland*

E-mail: sara.bonella@epfl.ch

1 P3MaZe evolution equations

We derive the Mass Zero equations of motion from the extended Lagrangian of Eq. (11) of the main text,

$$\begin{aligned}\mathcal{L}_{\mathrm{P3MaZe}} =& \frac{1}{2}\sum_{\beta=1}^{N_p} m_\beta \dot{\boldsymbol{r}}_\beta^T \dot{\boldsymbol{r}}_\beta + \frac{1}{2}\mu \dot{\boldsymbol{\phi}}_{\mathrm{LR}}^T \dot{\boldsymbol{\phi}}_{\mathrm{LR}} \\ & - \bar{U}(\{\boldsymbol{r}_\beta\}) - U_{\mathrm{SR}}(\{\boldsymbol{r}_\beta\}) - U_{\mathrm{LR}}(\boldsymbol{\phi}_{\mathrm{LR}};\{\boldsymbol{r}_\beta\}) - U_{\mathrm{self}} - \boldsymbol{\lambda}^T \boldsymbol{\sigma}_{\mathrm{LR}}(\boldsymbol{\phi}_{\mathrm{LR}};\{\boldsymbol{r}_\beta\}),\end{aligned} \quad (1)$$

where μ is the fictitious inertia of the auxiliary degrees of freedom $\boldsymbol{\phi}_{\mathrm{LR}}$, $\boldsymbol{\lambda}$ is the vector of Lagrange multipliers enforcing the constraint $\boldsymbol{\sigma}_{\mathrm{LR}} = \mathbf{0}$ [Eq. (13) of the main text], U_{LR} is the discretized long-range electrostatic energy [Eq. (12) of the main text], and U_{self} is the constant Gaussian self-energy defined in Eq. (7) of the main text. The Euler–Lagrange

equations are

$$
\begin{aligned}
m_\alpha \ddot{\boldsymbol{r}}_\alpha &= -\nabla_{\boldsymbol{r}_\alpha} U_{\mathrm{SR}}(\{\boldsymbol{r}_\beta\}) - \nabla_{\boldsymbol{r}_\alpha} U_{\mathrm{LR}}(\boldsymbol{\phi}_{\mathrm{LR}};\{\boldsymbol{r}_\beta\}) - \nabla_{\boldsymbol{r}_\alpha} \bar{U}(\{\boldsymbol{r}_\beta\}) \\
&\quad - \sum_n \lambda_n \nabla_{\boldsymbol{r}_\alpha} \sigma_{\mathrm{LR},n}(\boldsymbol{\phi}_{\mathrm{LR}};\{\boldsymbol{r}_\beta\}), \\
\mu\, \ddot{\phi}_{\mathrm{LR},n} &= -\frac{\partial U_{\mathrm{LR}}}{\partial \phi_{\mathrm{LR},n}} - \sum_m \lambda_m \frac{\partial \sigma_{\mathrm{LR},m}}{\partial \phi_{\mathrm{LR},n}}.
\end{aligned}
\tag{2}
$$

Along the trajectory, the constraints

$$
\sigma_{\mathrm{LR},n} = \frac{\partial U_{\mathrm{LR}}}{\partial \phi_{\mathrm{LR},n}} = \left(\frac{h}{4\pi} \boldsymbol{M} \boldsymbol{\phi}_{\mathrm{LR}} + \boldsymbol{q}^s \right)_n = 0 \tag{3}
$$

are satisfied so the force term $-\partial U_{\mathrm{LR}}/\partial \phi_{\mathrm{LR},n}$ in the equation for the auxiliary variables is null. We also have $\frac{\partial \sigma_{\mathrm{LR},m}}{\partial \phi_{\mathrm{LR},n}} = \frac{h}{4\pi} M_{mn}$. Using the symmetry of the matrix $\boldsymbol{M}$ and dividing by the inertia μ, we obtain for the evolution equation of the auxiliary variables

$$
\ddot{\boldsymbol{\phi}}_{\mathrm{LR}} = -\frac{h}{4\pi} \boldsymbol{M} \frac{\boldsymbol{\lambda}}{\mu}. \tag{4}
$$

We now take the limit $\mu \to 0$. For $\ddot{\boldsymbol{\phi}}_{\mathrm{LR}}$ to remain finite in this limit, we must have $\boldsymbol{\lambda} \to \boldsymbol{0}$ as $\mu \to 0$.[S1,S2] We therefore define the rescaled multiplier $\boldsymbol{\eta} = \lim_{\mu \to 0} \frac{h}{4\pi} \boldsymbol{\lambda}/\mu$, which remains finite in the zero–inertia limit for the auxiliary variables. With this definition, we have

$$
\ddot{\boldsymbol{\phi}}_{\mathrm{LR}} = -\boldsymbol{M} \boldsymbol{\eta}. \tag{5}
$$

Note that, for $\boldsymbol{\lambda} \to \boldsymbol{0}$, the constraint force term in Eq. (2) vanishes, and the equations of motion for the physical degrees of freedom reduce to

$$
m_\alpha \ddot{\boldsymbol{r}}_\alpha = -\nabla_{\boldsymbol{r}_\alpha} U_{\mathrm{SR}}(\{\boldsymbol{r}_\beta\}) - \nabla_{\boldsymbol{r}_\alpha} U_{\mathrm{LR}}(\boldsymbol{\phi}_{\mathrm{LR}};\{\boldsymbol{r}_\beta\}) - \nabla_{\boldsymbol{r}_\alpha} \bar{U}(\{\boldsymbol{r}_\beta\}), \tag{6}
$$

recovering exact physical dynamics and leading to Eq. (15) of the main text.

Numerical integration

The equations of motion above are integrated numerically using standard molecular dynamics algorithms.[S1,S2] The physical degrees of freedom $\boldsymbol{r}_\alpha$ are evolved via velocity Verlet (or OVRVO for (N, V, T) simulations), while the auxiliary variables $\boldsymbol{\phi}_{\mathrm{LR}}$ are propagated via the standard Verlet algorithm,

$$\boldsymbol{\phi}_{\mathrm{LR}}^{k+1} = 2\boldsymbol{\phi}_{\mathrm{LR}}^{k} - \boldsymbol{\phi}_{\mathrm{LR}}^{k-1} - \Delta t^2 \boldsymbol{M}\boldsymbol{\eta}, \tag{7}$$

where k denotes the time-step index and Δt is the timestep. Identifying the Verlet prediction $\boldsymbol{\phi}_{\mathrm{LR},p}^{k+1} = 2\boldsymbol{\phi}_{\mathrm{LR}}^{k} - \boldsymbol{\phi}_{\mathrm{LR}}^{k-1}$ as the provisional value, the time-updated field is

$$\boldsymbol{\phi}_{\mathrm{LR}}^{k+1} = \boldsymbol{\phi}_{\mathrm{LR},p}^{k+1} - \Delta t^2 \boldsymbol{M}\boldsymbol{\eta}. \tag{8}$$

The rescaled Lagrange multipliers $\boldsymbol{\eta}$ are determined at each step by enforcing $\boldsymbol{\sigma}_{\mathrm{LR}}(\boldsymbol{\phi}_{\mathrm{LR}}^{k+1}; \{\boldsymbol{r}_\alpha^{k+1}\}) = \boldsymbol{0}$. Substituting Eq. (8) into the constraint, we obtain

$$\frac{h}{4\pi}\boldsymbol{M}\boldsymbol{\phi}_{\mathrm{LR}}^{k+1} + \boldsymbol{q}^{s,k+1} = \boldsymbol{0} \implies \frac{h}{4\pi}\boldsymbol{M}\boldsymbol{\phi}_{\mathrm{LR},p}^{k+1} + \boldsymbol{q}^{s,k+1} = \frac{h}{4\pi}\Delta t^2 \boldsymbol{M}^2\boldsymbol{\eta}. \tag{9}$$

Defining $\boldsymbol{y}^{k+1} = \frac{h}{4\pi}\Delta t^2 \boldsymbol{M}\boldsymbol{\eta}^{k+1}$, the equation above reduces to

$$\boldsymbol{M}\boldsymbol{y}^{k+1} = \boldsymbol{\sigma}_{\mathrm{LR},p}^{k+1}, \tag{10}$$

which is the linear system of Eq. (20) of the main text. Once $\boldsymbol{y}^{k+1}$ is obtained, the updated field follows as

$$\boldsymbol{\phi}_{\mathrm{LR}}^{k+1} = \boldsymbol{\phi}_{\mathrm{LR},p}^{k+1} - \frac{4\pi}{h}\boldsymbol{y}^{k+1}. \tag{11}$$

This system is solved iteratively at each time step using the multigrid solver described in the main text. Since the provisional potential is constructed from previously gauge-fixed fields, it also has zero spatial mean. The gauge $\langle \phi_{\mathrm{LR}} \rangle = 0$ is therefore preserved by selecting

the zero-mean solution of the correction equation for $\boldsymbol{y}^{k+1}$. In practice, the spatial mean of $\boldsymbol{y}^{k+1}$ is removed during the correction step, and any residual mean is subtracted from the updated potential to prevent numerical drift. Solvability of the periodic Poisson equation additionally requires the mesh source to have zero spatial average. This condition is satisfied for the charge-neutral systems considered in this work. Systems with nonzero total particle charge can be treated by adding a uniform compensating background to the mesh source before constructing the Poisson constraint, as conventionally done in periodic Ewald and particle–mesh methods.

2 NVE stability and conservation properties

We assessed the NVE stability of P3MaZe using the SPC/Fw water system described in the main text. The system contains 216 water molecules in a cubic periodic cell of side 18.552 Å. All trajectories were started from the same equilibrated configuration at 298.15 K, propagated for 50 ps, and sampled every 10 fs. For P3MaZe, the total energy was evaluated both using the electrostatic work accumulated from the forces employed in the dynamics (see Eq. (25) of the main text) and the explicit estimator constructed from the long-range mesh term (Eq. (14)). Figure S1 compares the two quantities.

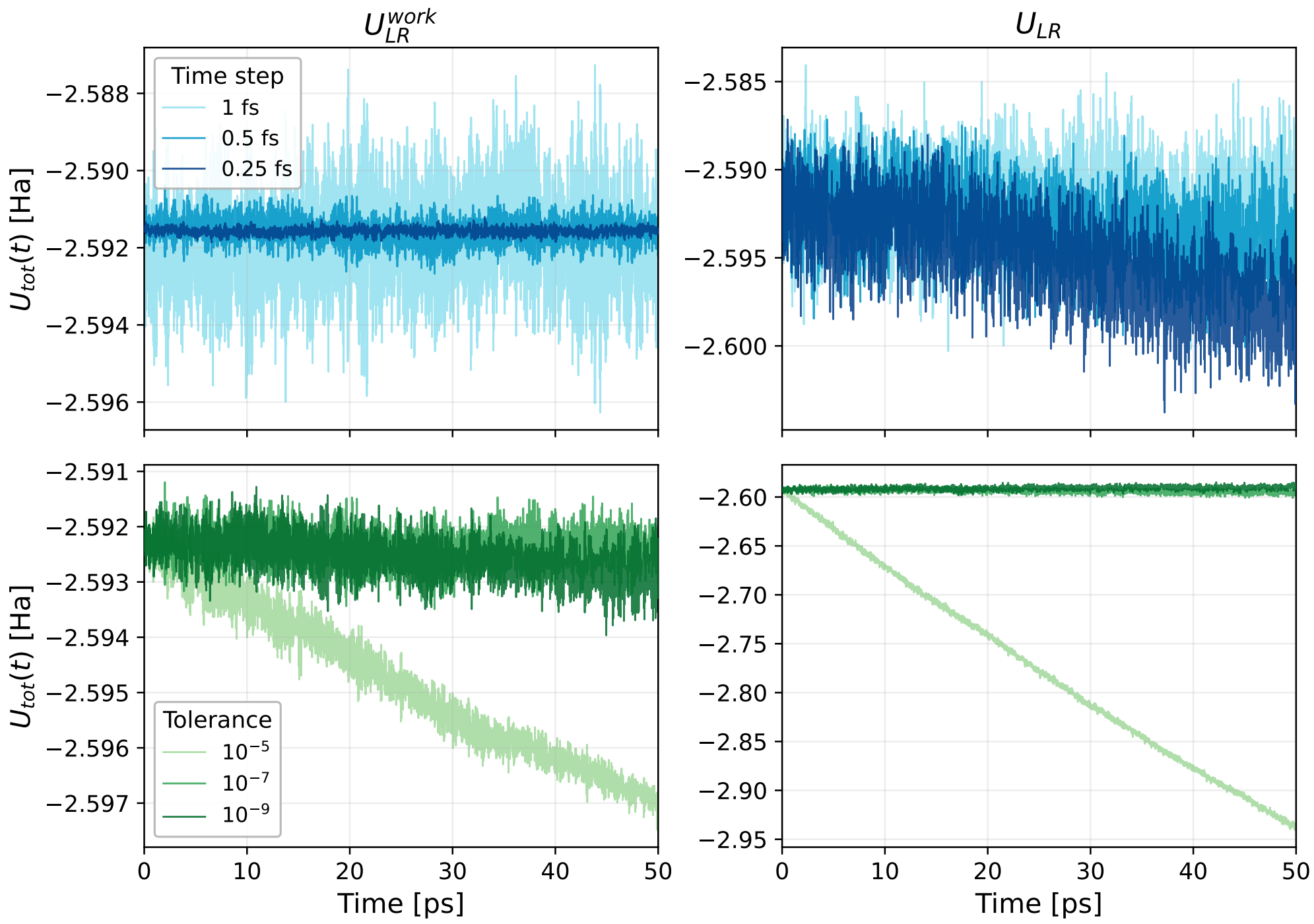

Figure S1: Comparison of the work-based and explicit total-energy estimators for SPC/Fw water simulated with P3MaZe. The upper panels show the time-step sweep at fixed constraint tolerance 10^{-7}, while the lower panels show the tolerance sweep at fixed time step 0.5 fs. The work-based estimator is consistent with the electrostatic forces employed in the dynamics and exhibits systematic convergence with both numerical parameters.

The work-based energy exhibits the expected improvement as the time step and constraint tolerance are reduced. By contrast, the explicit estimator retains finite-mesh fluctuations that do not decrease systematically with the time step. These fluctuations arise from the finite-grid representation of the mesh energy and its non-exact correspondence with the interpolated force. The analytical Gaussian self-energy is constant for fixed charges and screening width and therefore does not contribute to the observed drift. We therefore use $U_{\mathrm{LR}}^{\mathrm{work}}$ for the following NVE conservation analysis. As this estimator is reconstructed directly from the forces, however, its conservation is not an independent test of dynamical stability: by the work–energy theorem, changes in kinetic energy are compensated by opposite changes in the accumulated work, up to numerical integration and work-quadrature errors. A complementary and independent convergence analysis based directly on the kinetic energy, including

its dependence on the integration time step and solver tolerance, was reported for Poisson MaZe in Ref.[S2]

We first examine the dependence of the work-based NVE energy conservation on the integration time step. Simulations were performed with Δt = 1.0, 0.5, and 0.25 fs. The P3MaZe constraint tolerance was fixed at 10^{-7}. Equivalent trajectories were propagated using the P3M and Ewald implementations in LAMMPS, with the long-range accuracy fixed at 10^{-6}.[S3] Figure S2 reports the total-energy change and total-momentum norm for the three electrostatic methods. For all methods, the energy fluctuations decrease systematically as the time step is reduced. The relative fluctuations of the P3MaZe total energy decrease from 4.826×10^{-4} at 1 fs to 1.231×10^{-4} at 0.5 fs and 3.421×10^{-5} at 0.25 fs. The corresponding P3M and Ewald values are nearly identical, demonstrating that P3MaZe exhibits the same time-step convergence as the reference electrostatic methods.

The FFT-based P3M implementation and the Ewald solver evaluate the reciprocal-space contribution directly, without introducing an iterative-solver tolerance. Consequently, no residual iterative-solver error is present, and the total momentum is conserved close to machine precision in the present simulations. By contrast, real-space P3MaZe is iterative and the accuracy of its solution depends on the prescribed convergence tolerance. As shown below, tightening this tolerance systematically improves the conservation of the total momentum in our P3MaZe simulations, approaching the level observed for the direct reciprocal-space methods.

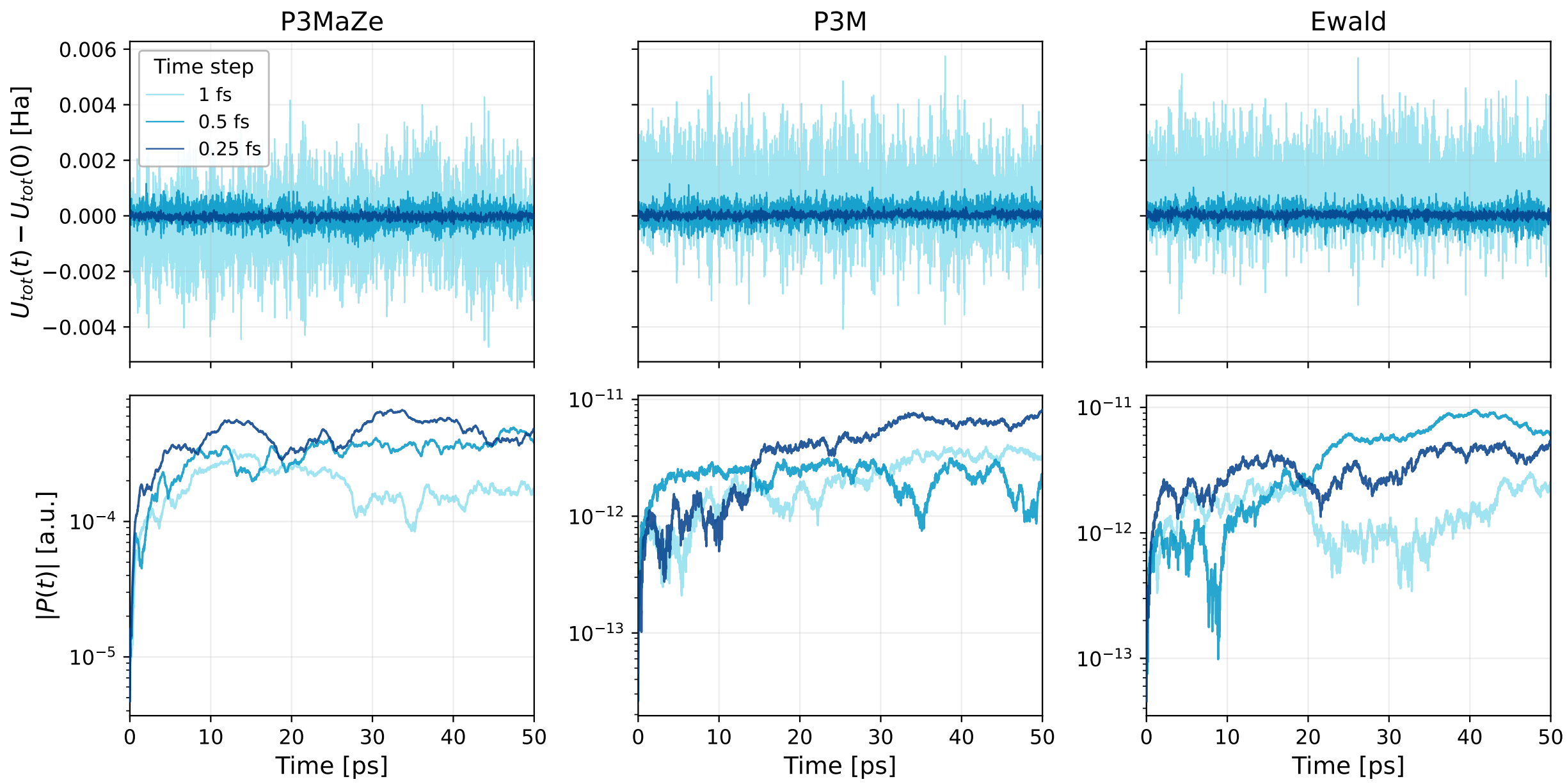

Figure S2: NVE stability of SPC/Fw water as a function of the integration time step for P3MaZe, P3M, and Ewald. The upper panels show the total-energy change relative to its initial value, while the lower panels show the norm of the total momentum. All trajectories span 50 ps. The energy fluctuations decrease systematically with Δt and are comparable among the three methods. No periodic momentum removal was applied during the trajectories.

We next examined the effect of the P3MaZe constraint tolerance at fixed $\Delta t = 0.5$ fs. Figure S3 compares tolerances of 10^{-5}, 10^{-7}, and 10^{-9}. A tolerance of 10^{-5} produces a visible systematic energy drift, whereas the trajectories obtained with 10^{-7} and 10^{-9} reduce or eliminate this drift over the 50 ps interval. The relative total-energy fluctuation decreases from 5.317×10^{-4} at 10^{-5} to 1.231×10^{-4} at 10^{-7}. Tightening the tolerance further to 10^{-9} gives a comparable fluctuation of 1.398×10^{-4}, indicating that further tightening of the constraint tolerance provides no measurable improvement at this integration time step.

Momentum conservation also improves systematically as the tolerance is tightened. This behavior shows that the residual momentum accumulation is associated with the finite accuracy of the constrained mesh solution and can therefore be controlled through the solver tolerance. Unlike other real-space approaches, our formulation does not require an explicit force correction to enforce total-momentum conservation. For example, in the real-space

multigrid method of Sagui and Darden,[S4] the force-interpolation procedure does not strictly conserve momentum, and the average net electrostatic force is explicitly subtracted from each particle at every MD step. No analogous force correction was applied in our P3MaZe simulations. The center-of-mass momentum was removed only once, at the beginning of each trajectory, and was not subsequently corrected during the dynamics. The reported momentum therefore reflects its free evolution from an initially zero-momentum state. As discussed in the section on force evaluation, the present formulation preserves total momentum in the converged solution without requiring an explicit correction.[S5]

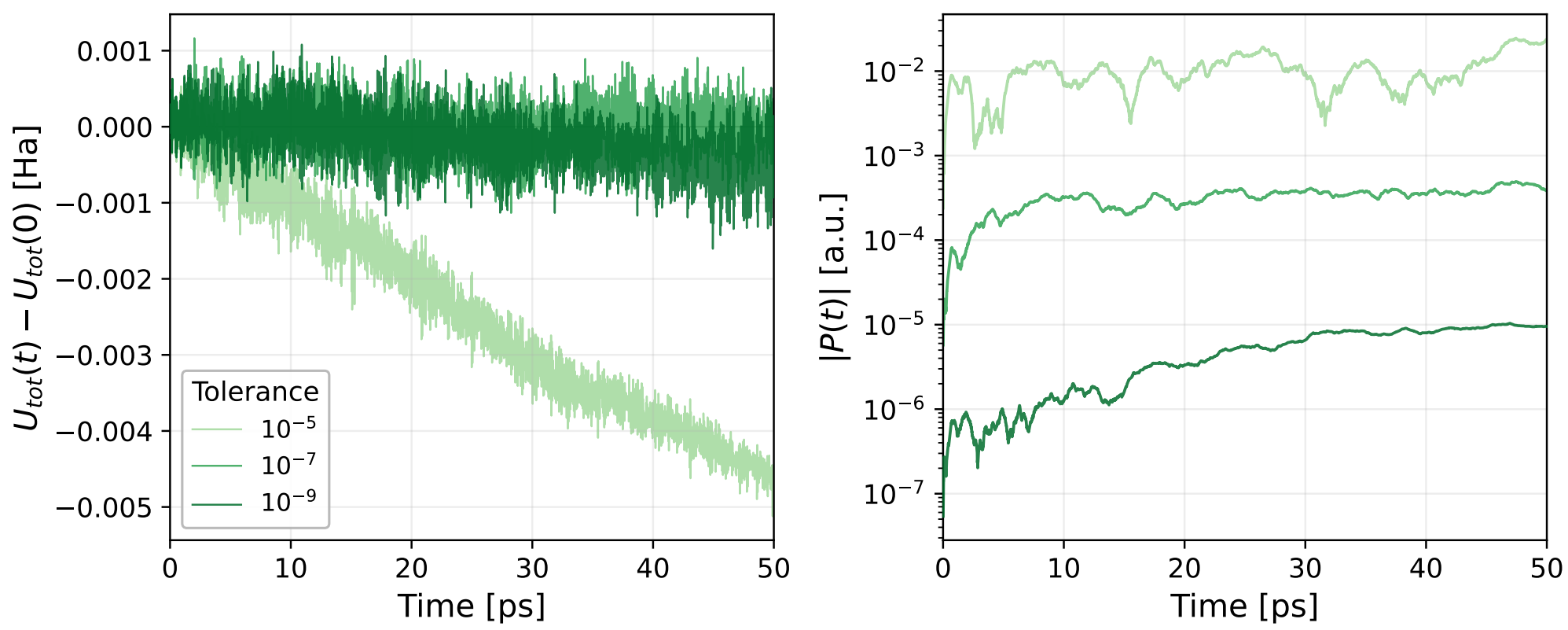

Figure S3: Dependence of the P3MaZe NVE stability on the constraint tolerance at fixed time step $\Delta t = 0.5$ fs. The left panel shows the total-energy change evaluated with the work-based estimator, while the right panel shows the total-momentum norm. Both energy and momentum conservation improve as the constraint tolerance is tightened.

The relative total- and potential-energy fluctuations are summarized in Table S1. We quantify the energy fluctuations through the relative standard deviations $\Delta U_{\mathrm{tot}}/|\langle U_{\mathrm{tot}}\rangle|$, and $\Delta U/|\langle U_{\mathrm{tot}}\rangle|$, where U_{tot} is the total energy, U is the total potential energy, and ΔU_{tot} and ΔU denote their sample standard deviations over the complete 50 ps trajectory. The time-step results show comparable total-energy fluctuations for P3MaZe, P3M, and Ewald. The tolerance sweep shows that a constraint tolerance of 10^{-7} is sufficient to make the P3MaZe energy fluctuations comparable to those of the reference methods at the same time step.

Table S1: Relative fluctuations of the total energy, $\Delta U_{\mathrm{tot}}/|\langle U_{\mathrm{tot}}\rangle|$, and potential energy, $\Delta U/|\langle U_{\mathrm{tot}}\rangle|$, for the NVE simulations of SPC/Fw water. For P3MaZe, the work-based and explicit $\frac{1}{2}q\phi$ energy definitions are reported separately; for P3M and Ewald, U is the total potential energy. The tolerance sweep is reported only for P3MaZe.

Method	Value	Energy definition	$\Delta U_{\mathrm{tot}}/|\langle U_{\mathrm{tot}}\rangle|$	$\Delta U/|\langle U_{\mathrm{tot}}\rangle|$
**Time step**				
P3MaZe	1 fs	E_{work}	4.826×10^{-4}	9.312×10^{-3}
P3MaZe	1 fs	$\frac{1}{2}q\phi$	8.468×10^{-4}	9.199×10^{-3}
P3M	1 fs	Total	4.867×10^{-4}	9.453×10^{-3}
Ewald	1 fs	Total	4.639×10^{-4}	9.372×10^{-3}
P3MaZe	0.5 fs	E_{work}	1.231×10^{-4}	9.289×10^{-3}
P3MaZe	0.5 fs	$\frac{1}{2}q\phi$	7.823×10^{-4}	9.162×10^{-3}
P3M	0.5 fs	Total	1.140×10^{-4}	9.172×10^{-3}
Ewald	0.5 fs	Total	1.133×10^{-4}	9.502×10^{-3}
P3MaZe	0.25 fs	E_{work}	3.421×10^{-5}	9.039×10^{-3}
P3MaZe	0.25 fs	$\frac{1}{2}q\phi$	9.963×10^{-4}	8.962×10^{-3}
P3M	0.25 fs	Total	3.402×10^{-5}	9.404×10^{-3}
Ewald	0.25 fs	Total	3.476×10^{-5}	9.256×10^{-3}
**Tolerance**				
P3MaZe	10^{-5}	E_{work}	5.317×10^{-4}	1.849×10^{-2}
P3MaZe	10^{-5}	$\frac{1}{2}q\phi$	3.588×10^{-2}	2.153×10^{-2}
P3MaZe	10^{-7}	E_{work}	1.231×10^{-4}	9.289×10^{-3}
P3MaZe	10^{-7}	$\frac{1}{2}q\phi$	7.823×10^{-4}	9.162×10^{-3}
P3MaZe	10^{-9}	E_{work}	1.398×10^{-4}	9.490×10^{-3}
P3MaZe	10^{-9}	$\frac{1}{2}q\phi$	7.316×10^{-4}	9.351×10^{-3}

As expected for NVE dynamics, the potential-energy fluctuations remain finite and nearly independent of the integration time step, whereas the total-energy fluctuations decrease systematically with the timestep.

3 Tuning of the P3MaZe parameters

The P3MaZe decomposition is controlled by the Gaussian screening width σ, the real-space cutoff R_c, and the number of mesh points per direction N. These parameters are coupled: narrower Gaussians require finer grids to resolve the screening charge density, but decay more rapidly in real space and therefore allow a smaller R_c.[S4] The production parameters were selected from explicit convergence tests on the molten-salt system, using as reference a highly converged Poisson MaZe calculation on a grid with $N = 380$ points per direction. For

fixed $\sigma = 1.39$ Å, the relative force error was computed for cutoffs in the range $R_c = 3.0$–8.0 Å (in steps of 0.5 Å). The error decreased monotonically with R_c and reached a plateau at approximately $R_c = 4.5$ Å, indicating that the residual error is no longer dominated by truncation of the short-range screened interaction. A convergence study in N at fixed σ and $R_c = 4.5$ Å then showed that $N = 60$ ($h = 0.344$ Å) yields a relative force error of approximately 2.5% with respect to the reference at moderate computational cost. These values were adopted for production simulations. The same analysis was performed for the SPC/Fw water model.

4 SPC/Fw parameters

Table adapted from.[S6]

Table S2: SPC/Fw model parameters adopted in this work, taken from Ref.[S6]

Parameter	Symbol	Value
Oxygen Lennard-Jones energy	ϵ_{OO}	0.1554253 kcal/mol
Oxygen Lennard-Jones size	σ_{OO}	3.165492 Å
Oxygen charge	q_{O}	$-0.82\,e$
Hydrogen charge	q_{H}	$+0.41\,e$
Equilibrium O–H bond length	r^0_{OH}	1.012 Å
Equilibrium H–O–H angle	θ^0_{HOH}	113.24°
Bond force constant	k_b	1059.162 kcal mol^{-1} Å^{-2}
Angle force constant	k_a	75.90 kcal mol^{-1} rad^{-2}

5 SPC/Fw rotational relaxation times

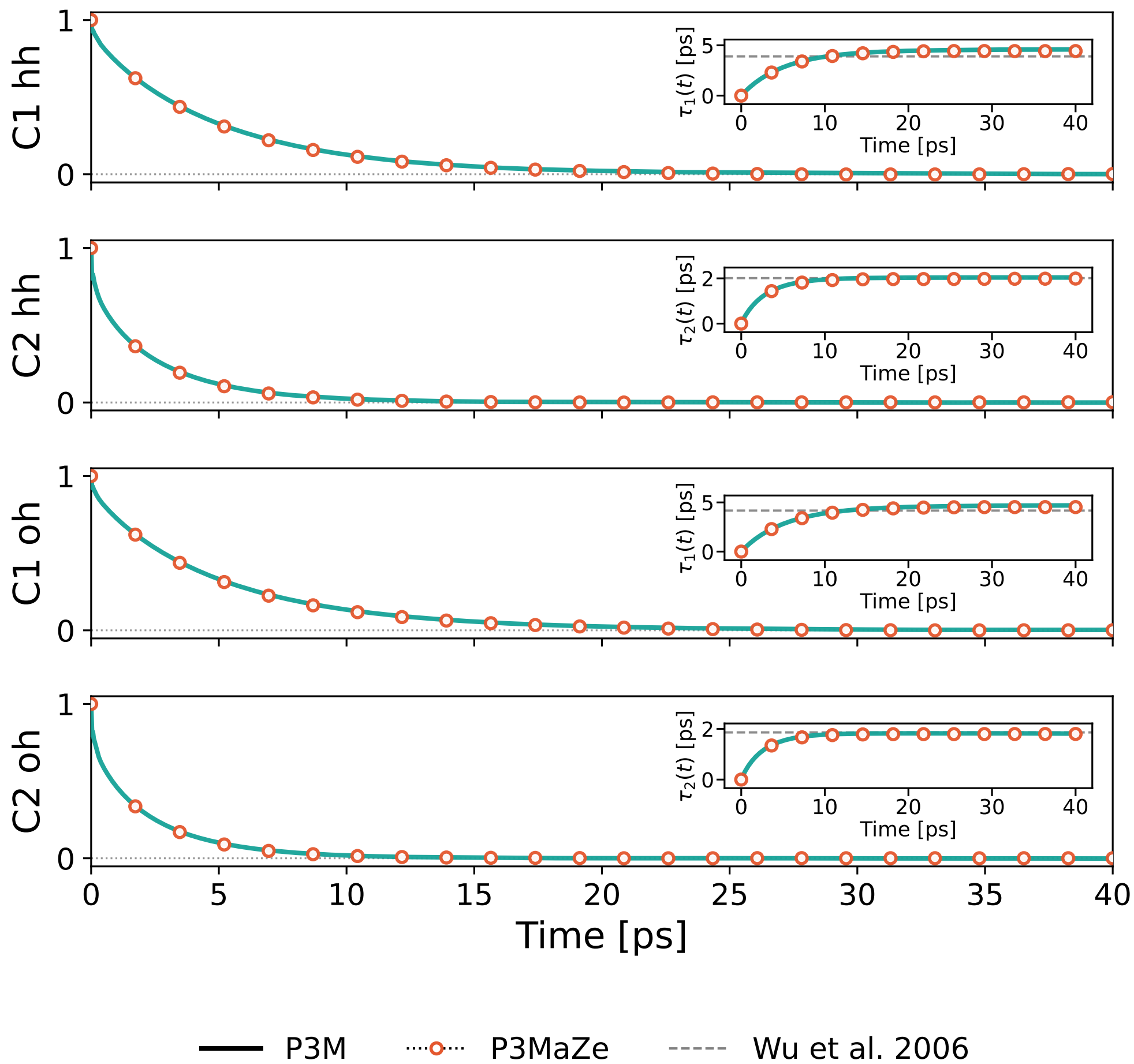

Figure S4: Comparison of rotational relaxation in SPC/Fw water for Particle-Particle Particle-Mesh (P3M) and P3MaZe, considering only the H–H and O–H molecular axes. For each observable, the main panels report the ensemble-averaged first- and second-order rotational correlation functions, while the insets show the corresponding cumulative integral estimates of the relaxation times $\tau_1(t)$ and $\tau_2(t)$. The horizontal dashed lines mark the reference values from Wu *et al.*[S6]

6 Direct comparison of the long-range electrostatic potential and its translational dependence

We performed a direct comparison of the long-range electrostatic potentials obtained with our in-house real-space Particle-Particle Particle-Mesh (P3M) implementation and P3MaZe,

using the equilibrated 250-ion molten NaCl configuration employed for the molten salt analysis. As shown in the main text, the P3MaZe correction problem and the direct P3M Poisson solve are algebraically equivalent representations of the same long-range mesh problem. Here, we provide an additional numerical verification of this equivalence by comparing the resulting electrostatic potentials and their dependence on rigid translations relative to the mesh. The ions were contained in a cubic box of side $L = 20.64$ Å. Both methods used the same 64^3 mesh, cubic B-spline charge assignment, Gaussian charge smoothing with $\sigma = 1.39$ Å, a real-space cutoff of 4.5 Å, and a solver tolerance of 10^{-7}. The 64^3 mesh was used specifically for this comparison; the molten NaCl production simulations used the 60^3 mesh reported in the main text. All calculations were performed with our in-house code to ensure consistent numerical treatment.

No molecular dynamics propagation was performed: the particle coordinates were frozen and a fresh solver instance was initialized for every calculation. The complete configuration was rigidly translated relative to the mesh along x, y, z, and the body diagonal. For each direction, 21 equally spaced positions were considered, spanning one mesh spacing in each translated Cartesian component and including the periodic endpoint. After each field update, we stored the vector $\boldsymbol{\phi}_{\mathrm{LR}}$ of mesh potentials, whose component $\phi_{\mathrm{LR},n}$ is the potential at grid point $\boldsymbol{r}_n$, with $n = 1, \ldots, N_g$ and $N_g = 64^3$. Because the electrostatic potential is defined up to an additive constant, the spatial mean was removed from every field. Translated fields were then registered to the unshifted coordinate system using periodic Fourier interpolation. The registered fields are used throughout the following comparison.

6.1 Distribution of the P3M–P3MaZe potential difference

At each mesh point, the registered potentials were averaged over 80 fields, comprising 20 samples from each of the four translation directions after excluding the periodic endpoint.

We then calculated the vector of potential differences

$$\Delta\boldsymbol{\phi}_{\mathrm{LR}}^{\mathrm{methods}} = \left\langle \boldsymbol{\phi}_{\mathrm{LR}}^{\mathrm{P3M}} \right\rangle_{\mathrm{shifts}} - \left\langle \boldsymbol{\phi}_{\mathrm{LR}}^{\mathrm{P3MaZe}} \right\rangle_{\mathrm{shifts}}. \tag{12}$$

Figure S5 shows the probability density of the components $\Delta\phi_{\mathrm{LR},n}^{\mathrm{methods}}$ over the N_g mesh points. The distribution is centred at zero, with a mean of -2.6×10^{-20} a.u. The RMS difference between the two fields averaged over translations is

$$\mathrm{RMS}\left(\Delta\boldsymbol{\phi}_{\mathrm{LR}}^{\mathrm{methods}}\right) = \left[\frac{1}{N_g}\sum_{n=1}^{N_g}\left(\Delta\phi_{\mathrm{LR},n}^{\mathrm{methods}}\right)^2\right]^{1/2} = 5.0 \times 10^{-8} \text{ a.u.}, \tag{13}$$

and the maximum absolute difference is 1.4×10^{-7} a.u. The distribution is not required to be Gaussian because its entries are spatially correlated values of a deterministic field rather than independent realizations of a random error. These values quantify the discrepancy between the two numerical methods, rather than an absolute error relative to an analytical reference.

6.2 Translation of the complete configuration relative to the mesh

For each method and a rigid translation $\boldsymbol{\Delta}$, we define the registered potential difference at grid point $\boldsymbol{r}_n$ as

$$\Delta\phi_{\mathrm{LR},n}(\boldsymbol{\Delta}) = \phi_{\mathrm{LR}}^{(\boldsymbol{\Delta})}(\boldsymbol{r}_n + \boldsymbol{\Delta}) - \phi_{\mathrm{LR}}^{(\mathbf{0})}(\boldsymbol{r}_n), \tag{14}$$

where $\phi_{\mathrm{LR}}^{(\boldsymbol{\Delta})}(\boldsymbol{r})$ denotes the potential reconstructed by periodic Fourier interpolation from the mesh solution for the translated configuration, after removal of its spatial mean. The vector $\Delta\boldsymbol{\phi}_{\mathrm{LR}}(\boldsymbol{\Delta})$ collects these pointwise differences. We quantify its magnitude through

$$\mathrm{RMS}\left(\Delta\boldsymbol{\phi}_{\mathrm{LR}}(\boldsymbol{\Delta})\right) = \left[\frac{1}{N_g}\sum_{n=1}^{N_g}\left(\Delta\phi_{\mathrm{LR},n}(\boldsymbol{\Delta})\right)^2\right]^{1/2}. \tag{15}$$

Figure S6 reports the RMS potential difference as a function of the translation compo-

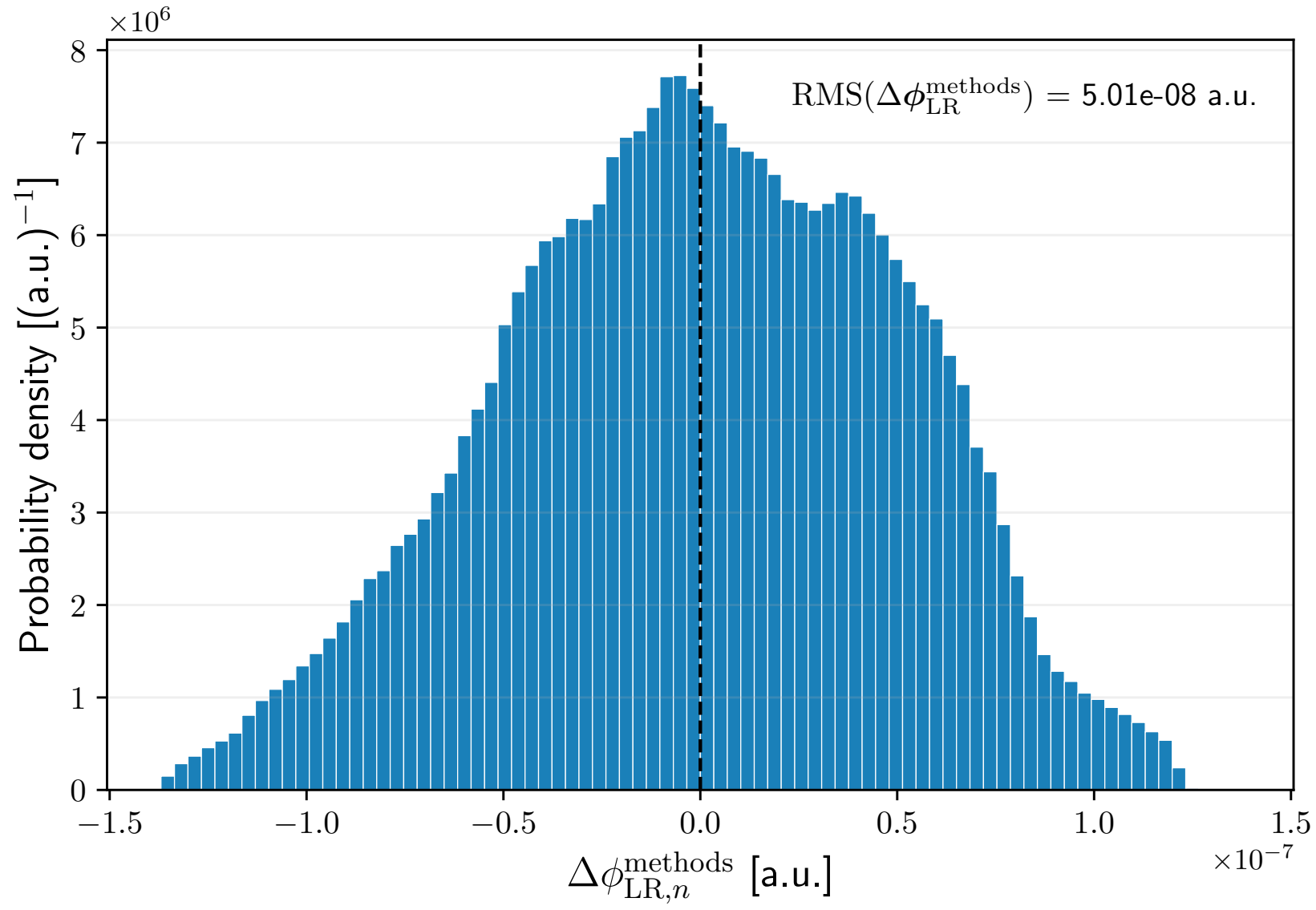

Figure S5: Probability density of the components $\Delta\phi_{\mathrm{LR},n}^{\mathrm{methods}}$ of the difference between the long-range potentials averaged over translations, evaluated over the 64^3 mesh points. The vertical dashed line denotes zero. The spatial mean was removed from each potential, and the fields were registered to the unshifted coordinate system before averaging over 80 fields, comprising 20 samples per translation direction after excluding the periodic endpoint.

nents in units of the mesh spacing h. For the diagonal scan, $\Delta x/h = \Delta y/h = \Delta z/h$. The P3M and P3MaZe curves are essentially indistinguishable. The RMS variation is zero for the unshifted configuration, is largest near a half-mesh translation, and returns to nearly zero after one complete mesh spacing in each translated component, as expected from mesh periodicity. Its maximum is approximately 7×10^{-7} a.u. for translations along the individual Cartesian directions and 1.23×10^{-6} a.u. for the body diagonal, where all three Cartesian components are varied simultaneously. The shaded region for P3M and the error bars for P3MaZe span the range between the minimum and maximum of $|\Delta\phi_{\mathrm{LR},n}(\mathbf{\Delta})|$ over the mesh points. They describe a spatial range and are not statistical confidence intervals from independent trajectories.

The RMS difference between the potentials averaged over translations is smaller than the maximum RMS variation induced by translations relative to the mesh. The agreement between the averaged fields and the coincident translational dependence are consistent with

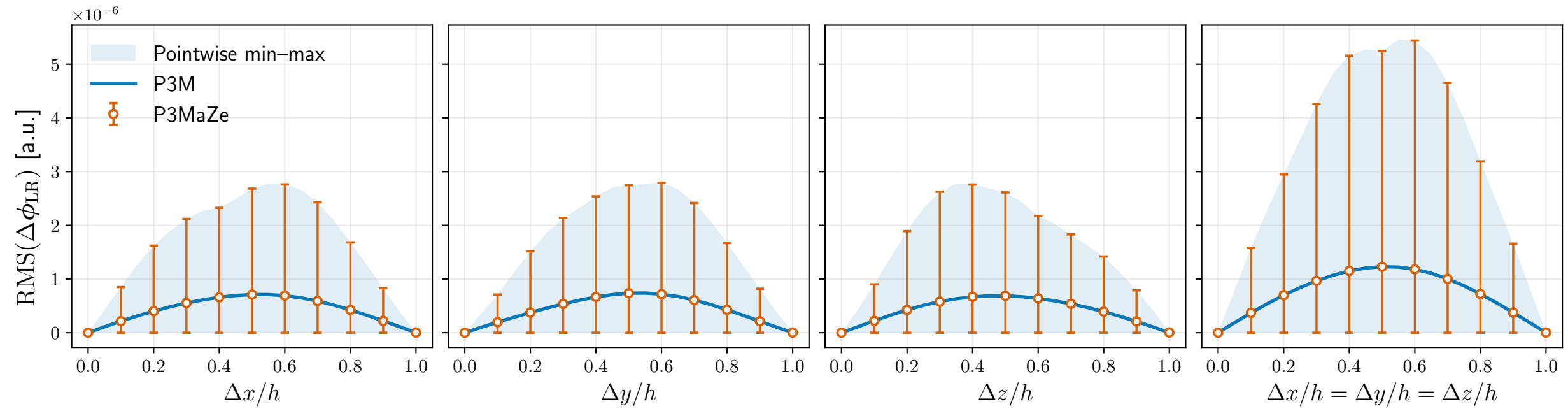

Figure S6: Translational dependence of the long-range electrostatic potential for P3M and P3MaZe. The ordinate is the RMS potential difference defined in Eq. (15). The abscissae are $\Delta x/h$, $\Delta y/h$, and $\Delta z/h$ for the Cartesian scans; for the body diagonal, $\Delta x/h = \Delta y/h = \Delta z/h$. A value of 1 corresponds to a translation of one mesh spacing along each indicated component. The shaded region and error bars span the range between the minimum and maximum of $|\Delta\phi_{\mathrm{LR},n}(\boldsymbol{\Delta})|$ over the mesh points.

the algebraic equivalence between the P3MaZe correction problem and the corresponding direct Poisson solve established in the main text.

7 Linear performance fits for Fig. 9

Table S3 reports the coefficients obtained by fitting the untransformed solver and total electrostatic CPU times per step as linear functions of the number of particles, $t = aN_p + b$. These fits complement the log–log representation shown in Fig. 9 of the main text: the slopes in the log–log representation characterize the scaling exponent, whereas the coefficients a_{solver} and a_{elec} quantify the increase in solver and total electrostatic cost, respectively, per additional particle.

Table S3: Slopes obtained from linear fits of the untransformed solver time, t_{solver}, and total electrostatic CPU time, t_{elec}, as functions of the number of particles N_p. All coefficients are reported in units of s. The ratios are normalized by the corresponding slope of P3MaZe with $\boldsymbol{y}_0 = \boldsymbol{y}_0^{(1)}$.

Method	a_{solver}	$a_{\text{solver}}/a_{\text{solver}}^{\text{P3MaZe}}$	a_{elec}	$a_{\text{elec}}/a_{\text{elec}}^{\text{P3MaZe}}$
**NaCl**				
P3MaZe - $\boldsymbol{y}_0 = \boldsymbol{y}_0^{(0)}$	2.076×10^{-4}	2.80	2.814×10^{-4}	1.78
P3MaZe - $\boldsymbol{y}_0 = \boldsymbol{y}_0^{(1)}$	7.423×10^{-5}	1.00	1.584×10^{-4}	1.00
Poisson MaZe	1.383×10^{-3}	18.63	1.387×10^{-3}	8.76
**SPC/Fw**				
P3MaZe - $\boldsymbol{y}_0 = \boldsymbol{y}_0^{(0)}$	2.627×10^{-5}	1.04	3.324×10^{-5}	1.03
P3MaZe - $\boldsymbol{y}_0 = \boldsymbol{y}_0^{(1)}$	2.518×10^{-5}	1.00	3.216×10^{-5}	1.00
P3MaZe - $\boldsymbol{y}_0 = \boldsymbol{y}_0^{(2)}$	1.722×10^{-5}	0.68	2.419×10^{-5}	0.75
P3MaZe - $\boldsymbol{y}_0 = \boldsymbol{y}_0^{(3)}$	1.514×10^{-5}	0.60	2.212×10^{-5}	0.69

The fitted coefficients a_{elec} quantify the increase in electrostatic cost per additional particle for each method and system. For SPC/Fw water, the total-cost ratio relative to P3MaZe with $\boldsymbol{y}_0 = \boldsymbol{y}_0^{(1)}$ decreases from 1.00 to 0.75 for $\boldsymbol{y}_0^{(2)}$ and 0.69 for $\boldsymbol{y}_0^{(3)}$. Thus, the higher-order predictors reduce the total cost per particle, although the incremental improvement becomes smaller with increasing predictor order. This diminishing benefit can be attributed partly to the smoothing overhead, whose contribution increases from approximately 13–18% for $\boldsymbol{y}_0^{(1)}$ to 24–26% for $\boldsymbol{y}_0^{(3)}$, thereby increasingly limiting the gains obtained by further improving the initial guess.

References

(S1) Coretti, A.; Bonella, S.; Ciccotti, G. Communication: Constrained molecular dynamics for polarizable models. *The Journal of Chemical Physics* **2018**, *149*, 191102.

(S2) Troni, F.; Grassano, D.; Narayan, J.; Roux, B.; Bonella, S. Mass-zero constrained molecular dynamics for electrostatic interactions. *The Journal of Chemical Physics* **2025**, *163*, 214106.

(S3) The LAMMPS long-range accuracy parameter is defined differently from the conver-

gence tolerance used in P3M-AZE; their numerical values are therefore not directly comparable.

(S4) Sagui, C.; Darden, T. Multigrid methods for classical molecular dynamics simulations of biomolecules. *The Journal of Chemical Physics* **2001**, *114*, 6578–6591.

(S5) Hockney, R. W.; Eastwood, J. W. *Computer Simulation Using Particles*; McGraw-Hill, 1981.

(S6) Wu, Y.; Tepper, H. L.; Voth, G. A. Flexible simple point-charge water model with improved liquid-state properties. *The Journal of Chemical Physics* **2006**, *124*.
